# DFT Studies on the Stereoselectivity of α-Silyloxy Diazoalkane Cycloadditions

**DOI:** 10.3390/molecules201219783

**Published:** 2015-12-02

**Authors:** Matthew J. O’Connor, Huaqing Liu, Daesung Lee, Tao Zhou, Yuanzhi Xia

**Affiliations:** 1 Department of Chemistry, University of Illinois at Chicago, 845 West Taylor Street, Chicago 60607, IL, USA; moconn26@uic.edu; 2 Abbvie Inc., 1 North Waukegan Road, North Chicago 60064, IL, USA; huaqing.liu@abbvie.com; 3 College of Chemistry and Materials Engineering, Wenzhou University, Wenzhou, Zhejiang 325035, China; xyz@wzu.edu.cn

**Keywords:** density functional theory, diazoalkane, [3+2] cycloaddition, gauche effect, stereoselectivity

## Abstract

The intramolecular [3+2] cycloaddition (32CA) of alkene-tethered α-silyloxydiazoalkanes provides variable stereoselectivity in generating bicyclic pyrazolines where the silyloxy group is either *syn* or *anti* to the newly formed pyrazoline ring. To elucidate the origin of the stereoselectivity, density functional theory (DFT) calculations were carried out for the energy of each transition state structure (TSs) and product. Steric effects were identified as the major determining factors in the diastereoselectivity of the 32CA reaction with regards to substrate structure (cyclic or acyclic α-silyloxydiazoalkanes).

## 1. Introduction

**Figure 1:**
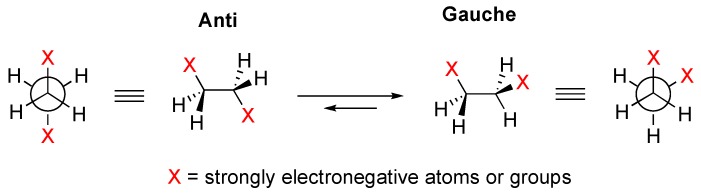
The gauche effect in 1,2-difluoro- and 1,2-dimethoxyetnane.

The gauche effect is a conformational phenomenon where two strongly electronegative 1,2-substituents, such as halogen-, oxygen-, and nitrogen-based functional groups, favor to assume the gauche over the corresponding anti conformation (Figure 1) [1,2,3,4,5,6].

One most representative example is the conformational behavior of 1,2-difluoroethane where the gauche conformation has a lower energy than the anti by 2.4−3.4 kJ/mol [7,8,9,10,11,12]. The gauche effect was also identified as a key factor in certain biological systems such as collagen [13,14,15,16]. Hyperconjugation and bent bonds-based explanations are proposed for the gauche effect, and the former is considered to be the principal cause of the preferred gauche conformation of 1,2-difluoro-ethane. In the hyperconjugation model, the maximum delocalization of the C–H electron density to the σ* orbital of the C–F bond will be achieved in the gauche conformation. This is because the C–H bond is a better electron donor than the C–F bond due to the greater electronegativity of fluorine, while the σ* orbital of the C–F bond is a better electron acceptor than that of the C–H bond.

In our recent study of the tandem addition of trimethylsilyldiazomethane (TMSD) to the carbonyl group of 4-alkenyl carbonyl compounds, followed by intramolecular [3+2] cycloaddition (32CA) of the diazoalkane intermediate with the tethered alkene to generate bi- and tricyclic Δ^1^-pyrazolines, an unusual stereochemical outcome trend was observed [17]. In this report, Lewis bases, such as potassium *tert*-butoxide (*t*-BuOK) or tetrabutylammonium triphenyldifluorosilicate (TBAT) catalyzed the tandem 1,2-addition, 1,3-Brook rearrangement followed by intramolecular 32CA of 4-alkenyl carbonyl compounds. For cyclic 4-alkenyl ketones, complete diastereoselectivity was observed in the reaction in favor of the *syn* adducts, as verified by X-ray crystallographic studies. When 4-alkenyl aldehydes were tested as substrates under the reaction conditions, the reaction was also selective, but favoring the *anti*-cycloadducts (2:1 to 1:0 dr). Finally, in the cases of 4-alkenyl ketones, the newly formed ∆^1^-pyrazolines were formed with low diastereoselectivity. To probe the origin of selectivity and either prove or refute the gauche effect hypothesis, DFT calculations were thus performed.

**Figure 2:**
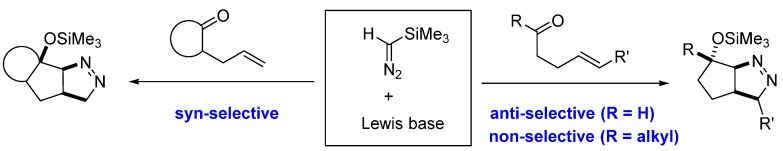
Selectivity in formation of ∆^1^-pyrazolines.

In the case of cyclic alkene-tethered α-silyloxydiazoalkanes, the seemingly less favorable diastereomer with *syn-*relationship of adjacent silyloxy group and C–N bond of the newly formed pyrazoline ring became more favorable over the corresponding *anti*-diastereomer. At a first glance, this seems to be the manifestation of the gauche effect of the vicinal C–O and C–N bonds. In the case of acyclic systems, steric effect overrides the gauche effect (Figure 2). To further elucidate the origin of the observed selectivity, the transition state structure (TSs) and product energies of representative systems containing different structural features were calculated.

## 2. Results and Discussion

### 2.1. DFT Calculations on Pyrazolines Formed with High syn-Diastereoselectivity

**Figure 3:**
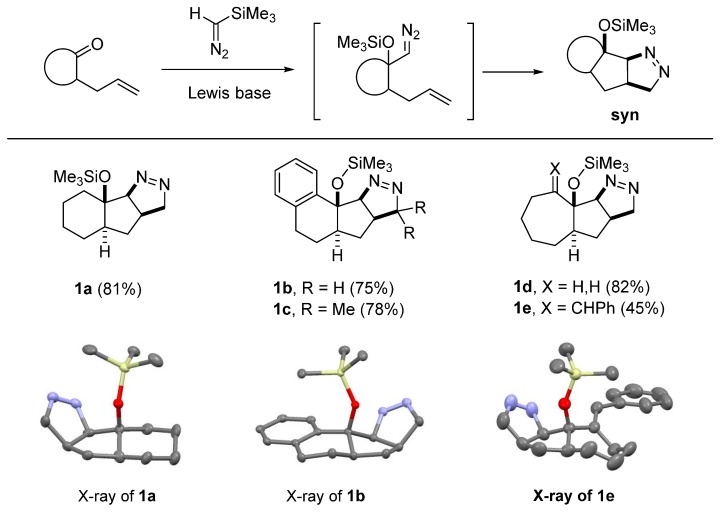
High *syn*-selectivity in the formation of α-silyloxypyrazolines.

It was recognized that pyrazolines **1a**–**e** formed as a single isomer upon treatment of α-allyl cyclic ketones with trimethylsilyldiazomethane (10 mol % *t*-BuOK, THF, 0 °C) (Figure 3) [17]. The structural confirmation, including X-ray crystallographic analysis of pyrazolines **1a**, **1b**, and **1e**, revealed that the newly formed pyrazoline ring is disposed *syn* to the trimethylsilyloxy group [18]. Intuitively, these C–O and C–N *syn*-disposed products are considered to be less favorable than the corresponding *anti*-diastereomers because of the lone-pair electrons on the oxygen and nitrogen moieties in close proximity. Careful examination of these structures indicates that the C–O bond of the silyloxy group and the C–N bond of the pyrazoline ring has a gauche relationship with dihedral angles about 54°. In the *anti*-diastereomers if they had been formed, these dihedral angles would be roughly 180°.

At this juncture, we suspected that the well-known gauche effect of the vicinal polar bonds such as C–O and C–N bonds could be a strong driving force in this system such that the 32CA are to occur favorably generating the observed *syn-*diastereomers over the *anti* ones. To explore the gauche effect and steric factors that cause the observed high *syn*-selectivity of 32CA, we carried out density functional theory calculations (B3LYP/6-31+G(d) level) in solution phase. In general, the energy of the TSs and products of the X-ray structure-based *syn-*diastereomers were obtained first, and then compared it with the energy of the optimized TSs and products structure of *anti-*diastereomers.

**Figure 4:**
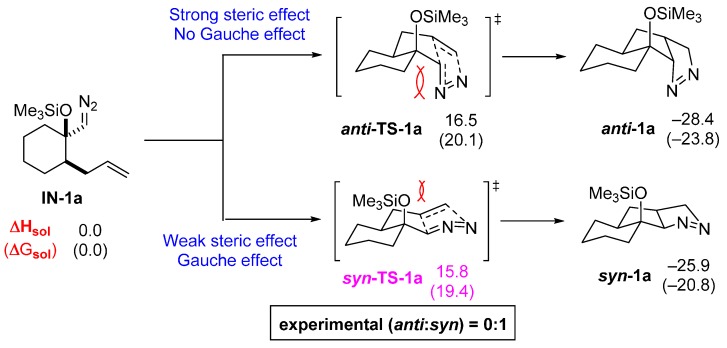
Energy profiles of pyrazoline formation with high *syn*-diastereoselectivity (in kcal/mol).

To gain insight into the role of the electronic (gauche effect) and steric factors for the formation of bicyclic pyrazoline **1a**, we used model intermediate **IN**-**1a** to compare the energies for the TSs and products of *syn*- and *anti*-isomers (Figure 4). It is expected that ***anti*-TS-1a** would have a strong steric effect but no gauche effect whereas the corresponding ***syn*-TS-1a** would manifest the expected gauche effect with only minimal steric effect. The calculated energy of these two TSs indeed correlate with the prediction such that the 32CA of diazoalkane **IN-1a** proceeds through ***syn*-TS-1a** that is 0.7 kcal/mol lower in energy compared to ***anti*-TS-1a**, generating the observed isomer ***syn*-1a**. This low DDE energy (0.7 kcal/mol) can be justified by the competing steric effects depicted in ***anti*-TS-1a**. Although the calculated energy of product ***anti*-1a** is lower than that of ***syn*-1a** by 2.5 kcal/mol, it is believed that this reaction is under kinetic control thus the formation of ***syn*-1a** through ***syn*-TS-1a** at relatively low temperature (0 °C) becomes a major pathway.

**Figure 5:**
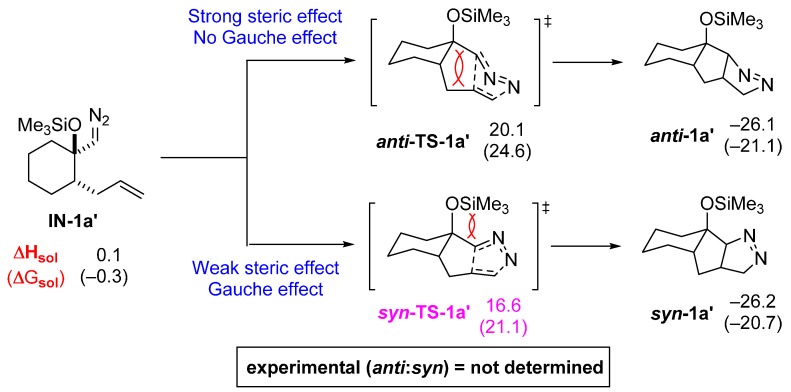
Energy profiles of pyrazoline formation with high *syn*-diastereoselectivity (in kcal/mol).

For comparison, we also calculated the energy profiles of isomer **IN-1a′** where the diazomethyl group and an allyl group have a *syn*-relationship, although this is not the isomer involved in the reaction (Figure 5). Even without calculation, we can predict that ***anti*-TS-1a′** will be sterically unfavorable compared to ***syn*-TS-1a′** because of their characteristic ring-junction stereochemistry. This prediction is verified by the calculation where ***syn*-TS-1a′** is 3.5 kcal/mol lower in energy.

### 2.2. DFT Calculations on Pyrazolines Formed with High Anti-Diastereoselectivity

**Figure 6:**
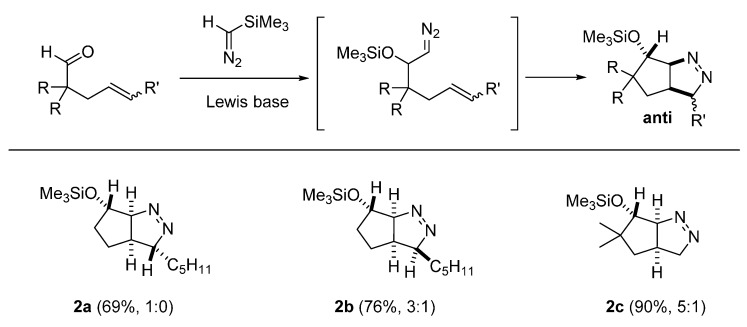
Tandem reaction of TMSD with 4-alkenyl aldehydes with high *anti*-diastereoselectivity.

Contrary to the 32CA of the diazo compound generated from cyclic ketones, when acyclic 4-alkenyl aldehydes were exposed to identical reaction conditions, the opposite preference of selectivity to form *anti*-diastereomers as exclusive or predominant products (Figure 6). The stereochemistry of these products was established by nuclear Overhauser effect (nOe) experiments, and further confirmed by converting **2a** to the corresponding *p*-nitrobenzoate followed by its X-ray diffractive analysis [18].

**Figure 7:**
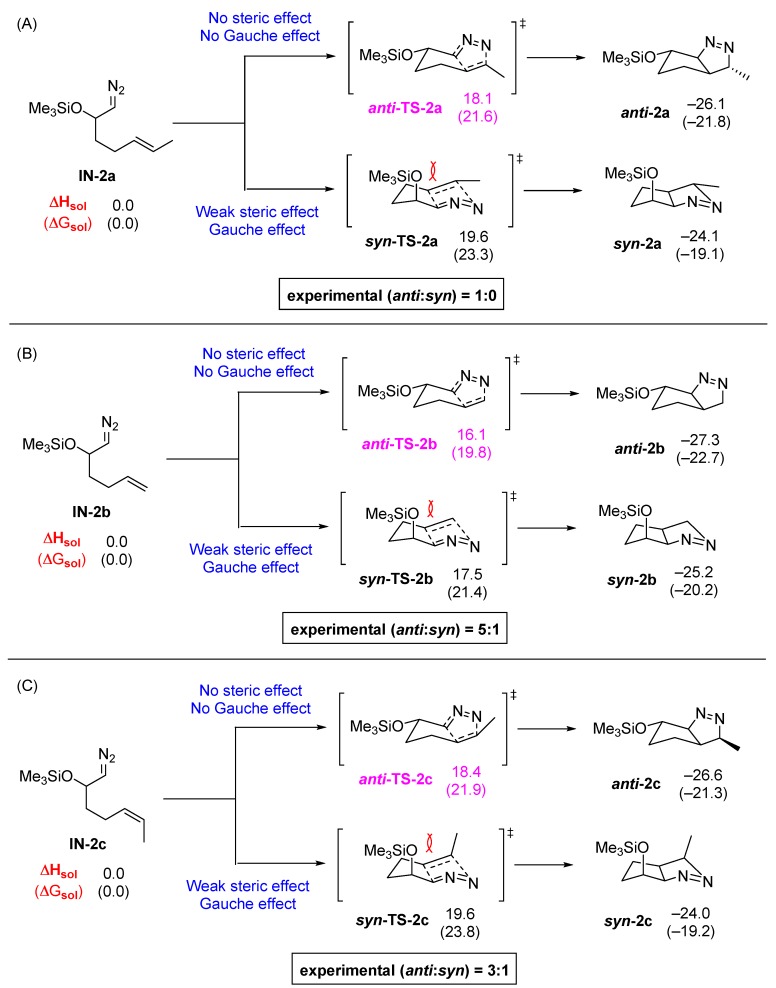
DFT calculations on pyrazolines formed with high *anti*-diastereoselectivity (in kcal/mol). (**A**) Energy profile for *E*-alkene; (**B**) Energy profile for terminal alkene; (**C**) Energy profile for *Z*-alkene.

To gain insight into the origin of the reversal of stereoselectivity for the formation of these *anti*-isomers, DFT calculations were carried out with **IN-2a** containing an internal *E*-alkene and **IN-2b** containing a terminal alkene (Figure 7). By simple examination, we predicted that the energy of TSs ***anti-*TS-2a** and ***syn*-TS-2a** (Figure 7A) as well as ***anti-*TS-2b** and ***syn*-TS-2b** (Figure 7B) would not be much different because the steric effect and the expected gauche effect are opposing each other. On the contrary, the DFT-calculated energy difference between ***anti*-TS-2a** and ***syn*-TS-2a** was calculated to be 1.5 kcal/mol and that of ***anti-*TS-2b** and ***syn*-TS-2b** was 1.4 kcal/mol. Considering the kinetically controlled nature of the reaction and the exclusive formation of bicyclic pyrazoline ***anti-*2a**, the calculated data is in good agreement with the observed experimental results. The favorable nature of ***anti*-TS-2a** and ***anti*-TS-2b** over the corresponding ***syn*-TS-2a** and ***syn*-TS-2b** suggest that in these systems, the steric effect plays a more important role than the gauche effect. For comparison, the energy profile of the 32CA of a *Z*-alkene containing system **IN-2c** was calculated (Figure 7C). The overall reaction profile of **IN-2c** is almost identical to that of the corresponding *E*-alkene-containing **IN-2a**, which implies that the *E*- or *Z*-alkyl configuration difference in the alkene does not have a significant steric influence for the energy of the TSs or the final products.

### 2.3. DFT Calculations on Pyrazolines Formed with Negligible Diastereoselectivity

**Figure 8:**
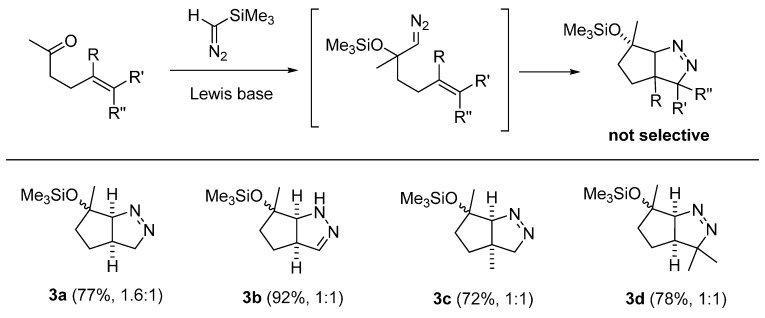
Pyrazolines formed with low diastereoselectivity.

Finally, DFT studies on the 32CA of α-*tert*-silyloxydiazo compounds generated from 4-alkenyl ketones were carried out. Different from the high to good selectivity for the 32CA of cyclic ketone- or aldehyde-derived diazo compounds, these acyclic ketone-derived systems provided pyrazoline products with no or negligible selectivity (Figure 8). The low selectivity for the formation of these pyrazolines is not unexpected, because it was already shown that even a small change in steric factors can affect the energy of the TSs, which would lead to the change is product selectivity (Figure 4, Figure 5 and Figure 7).

**Figure 9:**
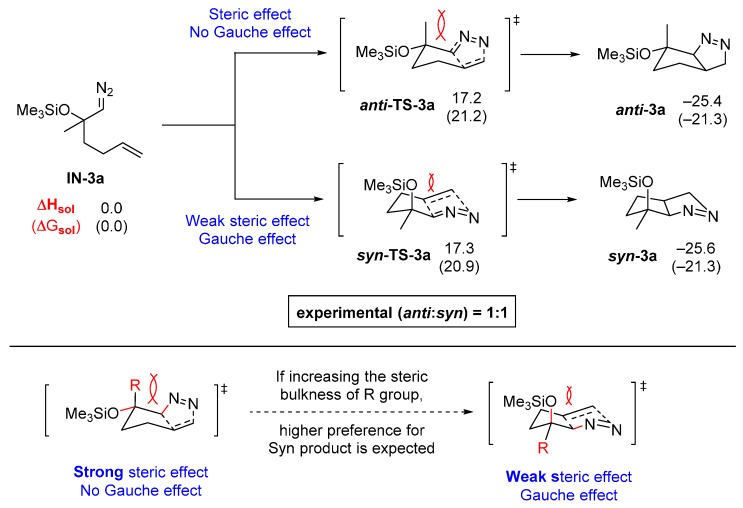
DFT calculations on pyrazolines formed with low diastereoselectivity (in kcal/mol).

To justify these outcomes, we assessed the energy difference between the two TSs ***anti*-TS-3a** and ***syn*-TS-3a** formed from intermediate **1N-3a** and the corresponding cycloadducts ***anti*-3a** and ***syn*-3a** (Figure 9). As shown in the calculated results, the energy difference between ***anti*-TS-3a** and ***syn*-TS-3a** is only 0.1 kcal/mol, which suggests that the selectivity for the formation of ***anti*-3a** and ***syn*-3a** should be negligible or low at best. This is indeed that case for **3a**–**3d**, which are generated from the 4-alkenyl acyclic ketones with little or no diastereoselectivity.

In comparison with the energy profile for the reaction of **IN-2b** containing a secondary α-silyloxy substituent, the calculated data for **IN-3a**, containing a tertiary α-silyloxy group with additional methyl group, clearly suggests that the increased steric bulk by the axial methyl substituent in ***anti*-TS-3a** destabilizes this TSs compared to ***anti*-TS-2b** in Figure 7, thus the selectivity decreased.

## 3. Experimental Section

### 3.1. Calculation Details

All calculations were carried out with the Gaussian 09 suite of computational programs [19]. The geometry optimizations were done at the B3LYP/6-31+G(d) level of theory [20,21,22]. Frequencies were analytically computed at the same level of theory to obtain the enthalpies and free energies and to confirm whether the structures are minima (no imaginary frequency) or TSs (only one imaginary frequency). The effect of THF solvent was included in all optimizations by using the PCM model with the default UFF atomic radii. Unless stated otherwise, all the energy values discussed in the main text are relative enthalpies (ΔH_sol_) in kcal/mol, and the relative free energies (ΔG_sol_) are given in related figures for reference. Only the intermediate or TSs that has the lowest energy value among all possible conformers is used for discussion.

### 3.2. General Procedure for the Preparation of Pyrazolines

To a stirred solution of carbonyl compound (1 mmol) in anhydrous THF (5 mL) under an atmosphere of nitrogen was added trimethylsilyldiazomethane (0.55 mL, 2.0 M in ether, 1.1 mmol) at 0 °C. The appropriate catalyst (TBAT or KO*t*Bu) was added, and the reaction was monitored by TLC. The reaction mixture was quenched with several drops of saturated aqueous solution of NH_4_Cl, and then dried over MgSO_4_. The drying reagent was filtered and solvent was removed under reduced pressure to give the crude product. Subsequent purification using flash chromatography (gradient elusion, hexane-ethyl acetate, 1:0→6:1) to afford the pure pyrazoline. Full characterization data can be found in the supplementary materials.

## 4. Conclusions

In summary, we have explored the origin of stereoselectivity for the intramolecular dipolar 32CA of diazoalkane species to form ∆^1^-pyrazolines by DFT calculations. In the case of diazo compounds derived from α-allyl cyclic ketones, the high C–O and C–N *syn*-selectivity in generating pyrazolines can be justified by the gauche effect, whereby the C–O bond of the silyloxy group and the C–N bond of the pyrazoline favor a gauche relationship, but this effect could be very weak in the reaction transition state. More likely, the stereoselectivity is merely the consequence of the steric effect of the fused ring-junction of the tricyclic systems and the gauche conformation is a result of this. Although the gauche effect has been identified as an electronic factor that influence the conformational behaviors of flexible acyclic systems [1], our study shows that the gauche effect is not an effective handle to control the stereoselectivity of the diazo compound-based 32CA in the acyclic systems examined herein, while in the examined cyclic systems, the observed gauche conformation of C–O and C–N bonds may merely be the result of a predominant steric effect of these particular frameworks. Only modest diastereoselectivity was observed for 4-alkenyl aldehydes, and no selectivity for the corresponding ketones. The selectivity trend in these systems can be best justified by kinetically controlled mode of reaction, where the steric effect and a possible gauche effect contribute to the respective TSs.

## Supplementary Materials

Supplementary materials can be accessed at: http://www.mdpi.com/1420-3049/20/12/19783/s1.
