# Supplementary material for: DFT Studies on the Stereoselectivity of α-Silyloxy Diazoalkane Cycloadditions
Source: Molecules. 2015 Dec 2;20(12):21433–41. doi: 10.3390/molecules201219783 (PMC6331888; doi:10.3390/molecules201219783)
Supplement: Supplementary file 1 [file molecules-20-19783-s001.pdf]

Supplementary Materials: DFT Studies on the Stereoselectivity of α-Silyloxy Diazoalkane Cycloadditions

Matthew J. O'Connor, Huaqing Liu, Daesung Lee, Tao Zhou and Yuanzhi Xia

Table of Contents

1. General Information S2
2. General Procedure for Preparation of Pyrazolines S2
3. Characterization Data S3
4. Computational Data S6
5. ^{1}H-NMR and ^{13}C-NMR Spectra S14
6. ORTEP Diagrams of Compounds **1a, 1b, 1e, PNB-2a** S28
7. Geometric TSs with Bond Lengths S29

1. General Information

1.1. Calculation Details

All calculations were carried out with the Gaussian 09 suite of computational programs. The geometry optimizations were done at the B3LYP/6-31+G(d) level of theory. Frequencies were analytically computed at the same level of theory to obtain the enthalpies and free energies and to confirm whether the structures are minima (no imaginary frequency) or transition states (only one imaginary frequency). The effect of THF solvent was included in all optimizations by using the PCM model with the default UFF atomic radii. Unless stated otherwise, all the energy values discussed in the main text are relative enthalpies (ΔH_{sol}) in kcal/mol, and the relative free energies (ΔG_{sol}) are given in related figures for reference. Only the intermediate or transition state that has the lowest energy value among all possible conformers is used for discussion.

1.2. Experimental Details

All reactions were carried out under an inert nitrogen atmosphere, unless otherwise indicated. Flasks were oven-dried overnight and cooled under a stream of nitrogen. Compounds were purchased from Aldrich unless otherwise noted. CH_2Cl_2, THF, Et_2O were purified based on standard procedures. Flash chromatography was performed using silica gel 60 Å (32–63 mesh) purchased from Sorbent Technologies. Analytical thin layer chromatography (TLC) was performed on 0.25 mm E. Merck precoated silica gel 60 (particle size 0.040–0.063 mm). ^1H NMR and ^{13}C NMR spectra were recorded on a Bruker DRX-500 spectrometer. ^1H and ^{13}C chemical shifts were referenced to internal solvent resonances and reported relative to $SiMe_4$; multiplicities are indicated by s (singlet), d (doublet), t (triplet), q (quartet), qn (quintet), m (multiplet) and br (broad). Coupling constants, *J*, are reported in Hz (Hertz). Electrospray ionization (ESI) mass spectra were recorded on a Micromass LCT equipped with a time-of-flight analyzer on a Waters Micromass Q-Tof Ultima in the University of Illinois at Urbana-Champaign. Electron impact (EI) mass spectra were obtained using a Micromass AutoSpecTM. Fast atom bombardment (FAB) mass spectra were taken at the Mass Spectrometry Laboratory in the University of Illinois at Urbana-Champaign, using a Micromass 70-VS-4F and 70-VSE for HRFAB and LRFAB, respectively. IR spectra were recorded using ATI Mattson, Genesis series FTIR. Optical rotations were measured on a JASCO DIP-370 digital polarimeter.

2. General Procedure for the Preparation of Δ¹-Pyrazolines

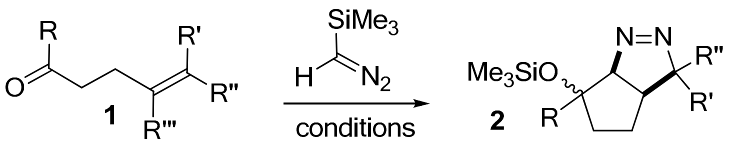

To a stirred solution of carbonyl compound (1 mmol) in anhydrous THF (5 mL) under an atmosphere of nitrogen was added trimethylsilyldiazomethane (0.55 mL, 2.0 M in ether, 1.1 mmol). The appropriate catalyst was added, and the reaction was monitored by TLC. The reaction mixture was quenched with several drops of saturated aqueous solution of NH_4Cl, and then dried over $MgSO_4$. The drying reagent was filtered and solvent was removed under reduced pressure to give the crude product. Subsequent purification using flash chromatography (gradient elusion, hexane:ethyl acetate, 1:0→6:1) to afford the pure pyrazoline **2**.

3. Characterization Data

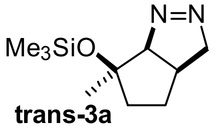

trans-3a

^{1}H-NMR (500 MHz, $CDCL_3$) δ 4.75–4.60 (m, 1H), 4.47 (ddd, *J* = 18.3, 9.2, 2.2 Hz, 1H), 4.33 (dt, *J* = 18.3, 3.0 Hz, 1H), 2.51 (q, *J* = 8.9 Hz, 1H), 2.13–1.92 (m, 1H), 1.66 (s, 3H), 1.62–1.49 (m, 1H), 1.27–1.12 (m, 1H), 0.83 (td, *J* = 12.9, 7.4 Hz, 1H), 0.23–0.11 (m, 9H). ^{13}C-NMR (101 MHz, $CDCL_3$) δ 103.85, 85.51, 83.07, 37.97, 32.48, 31.13, 24.54, 2.24. HRMS (ESI) calc. for $C_{10}H_{21}N_2OSi$ $[M + H]^+$ 213.1423, found 213.1421.

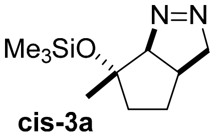

cis-3a

^{1}H-NMR (400 MHz, $CDCL_3$) δ 4.71 (dd, *J* = 7.5, 1.1 Hz, 1H), 4.51 (ddd, *J* = 17.9, 9.7, 1.4 Hz, 1H), 4.22–4.12 (m, 1H), 2.39–2.26 (m, 1H), 1.79 (tdt, *J* = 11.5, 7.2, 5.7 Hz, 1H), 1.56 (s, 3H), 1.51 (dd, *J* = 13.0, 6.3 Hz, 1H), 1.48–1.40 (m, 1H), 1.36–1.27 (m, 1H), 0.11 (s, 9H). ^{13}C-NMR (101 MHz, $CDCL_3$) δ 101.18, 84.99, 82.03, 40.07, 32.74, 29.89, 27.17, 2.19. HRMS (ESI) calc. for $C_{10}H_{21}N_2OSi$ $[M + H]^+$ 213.1418, found 213.1421.

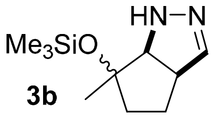

3b

^{1}H-NMR (500 MHz, $CDCl_3$) δ 6.58 (d, *J* = 1.43 Hz, 1H, major), 6.48 (s, 1H, minor), 3.74 (d, *J* = 1.43 Hz), 3.54 (t, *J* = 9.68, 9.68 Hz), 3.47–3.41 (m), 2.09–2.00 (m), 1.76–1.63 (m), 1.57–1.52 (m), 1.49–1.43 (m), 1.41–1.34 (m), 1.37 (s, 3H minor), 1.25 (s, 3H, major), 0.12 (s, 9H, minor), 0.10 (s, 9H, major). ^{13}C-NMR (125 MHz, $CDCl_3$) δ 147.80, 146.56, 85.22, 82.76, 77.28, 72.79, 68.12, 51.01, 50.13, 37.92, 28.59, 28.26, 27.85, 22.86, 2.38, 2.19. HRMS (ESI) calc. for $C_{10}H_{21}N_2OSi$ $[M + H]^+$ 213.1423, found 213.1423.

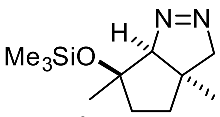

trans-3c

^{1}H-NMR (500 MHz, $CDCL_3$) δ 4.37 (dt, *J* = 10.9, 5.4 Hz, 1H), 4.29–4.18 (m, 2H), 1.87–1.73 (m, 1H), 1.65–1.60 (m, 1H), 1.59 (d, *J* = 4.5 Hz, 3H), 1.45 (dd, *J* = 12.4, 7.1 Hz, 1H), 1.19 (s, 3H), 0.89 (ddd, *J* = 21.0, 13.5, 6.8 Hz, 1H), 0.20–0.09 (m, 9H). ^{13}C-NMR (126 MHz, $CDCL_3$) δ 108.21, 92.05, 83.33, 41.37, 38.99, 38.70, 26.50, 24.55, 2.25. HRMS (ESI) calc. for $C_{11}H_{23}N_2OSi$ $[M + H]^+$ 227.1580, found 227.1590.

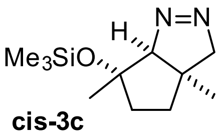

cis-3c

^{1}H-NMR (500 MHz, $CDCL_3$) δ 4.34 (t, *J* = 3.9 Hz, 1H), 4.31 (d, *J* = 17.9 Hz, 1H), 4.12 (dd, *J* = 17.9, 2.9 Hz, 1H), 1.72–1.62 (m, 1H), 1.60–1.57 (m, 1H), 1.55 (dd, *J* = 11.7, 5.7 Hz, 1H), 1.50–1.43 (m, 1H), 1.03 (s, 1H), 0.10–0.05 (m, 1H). ^{13}C-NMR (126 MHz, $CDCL_3$) δ 107.10, 91.54, 82.56, 41.50, 40.96, 37.04, 27.01, 26.87, 2.12. HRMS (ESI) calc. for $C_{11}H_{23}N_2OSi$ $[M + H]^+$ 227.1580, found 227.1587.

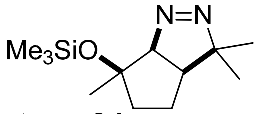

trans-3d

^{1}H-NMR (500 MHz, $CDCl_3$) δ 4.71 (d, *J* = 7.09 Hz, 1H), 2.20 (t, *J* = 8.27, 1H), 1.82–1.74 (m, 1H), 1.70 (s, 3H), 1.54 (dd, *J* = 12.88, 6.66 Hz, 1H), 1.45 (s, 3H), 1.45–1.40 (m, 1H), 1.15 (s, 3H), 0.85 (dt, *J* = 12.71, 12.69, 7.24 Hz, 1H), 0.16 (s, 9H). ^{13}C-NMR (125 MHz, $CDCl_3$) δ 104.19, 90.39, 83.39, 43.94, 39.01, 27.61, 25.08, 24.60, 21.69, 2.39. HRMS (ESI) calc. for $C_{12}H_{25}N_2OSi$ $[M + H]^+$ 241.1736, found 241.1736.

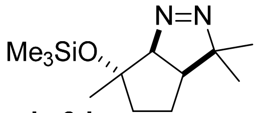

cis-3d

^{1}H-NMR (500 MHz, $CDCl_3$) δ 4.72 (d, *J* = 8.47 Hz, 1H), 2.00 (q, *J* = 13.24, 8.41 Hz, 1H), 1.56 (s, 3H), 1.54–1.45 (m, 4H), 1.40 (s, 3H), 1.19 (s, 3H), 0.14 (s, 9H). ^{13}C-NMR (125 MHz, $CDCl_3$) δ 101.96, 89.74, 81.82, 43.57, 40.72, 28.21, 27.44, 24.14, 21.42, 2.42. HRMS (ESI) calc. for $C_{12}H_{25}N_2OSi$ $[M + H]^+$ 241.1736, found 241.1736.

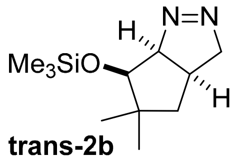

trans-2b

^{1}H-NMR (500 MHz, CDCL$_3$) δ 4.84–4.72 (m, 1H), 4.38–4.20 (m, 2H), 3.49 (t, *J* = 13.2 Hz, 1H), 2.46 (pd, *J* = 9.2, 3.1 Hz, 1H), 1.83–1.70 (m, 1H), 0.92 (d, *J* = 7.7 Hz, 3H), 0.89–0.83 (m, 4H), 0.74 (dd, *J* = 12.8, 10.1 Hz, 2H), 0.17 (s, 9H). ^{13}C-NMR (126 MHz, CDCL$_3$) δ 102.54, 82.95, 82.32, 44.51, 42.71, 30.67, 25.93, 20.74, 0.22. HRMS (ESI) calc. for $C_{11}H_{23}N_2OSi$ $[M + H]^+$ 227.1580, found 227.1587.

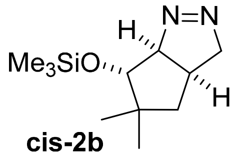

cis-2b

^{1}H-NMR (500 MHz, CDCL$_3$) δ 5.32 (dt, *J* = 8.5, 3.9 Hz, 1H), 4.54 (ddd, *J* = 15.5, 7.8, 2.7 Hz, 1H), 4.05 (t, *J* = 5.4 Hz, 1H), 4.01–3.92 (m, 1H), 2.53–2.23 (m, 1H), 1.70–1.49 (m, 1H), 1.39–1.16 (m, 2H), 0.92 (d, *J* = 6.6 Hz, 4H), 0.85 (d, *J* = 6.4 Hz, 4H), 0.08 (d, *J* = 3.2 Hz, 9H). ^{13}C-NMR (126 MHz, CDCL$_3$) δ 97.84, 83.42, 80.00, 77.25, 76.99, 76.74, 46.18, 43.55, 32.52, 25.46, 22.35. HRMS (ESI) calc. for $C_{11}H_{23}N_2OSi$ $[M + H]^+$ 227.1580, found 227.1587.

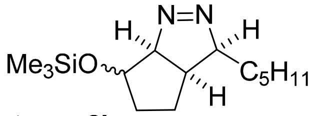

trans-2b

^{1}H-NMR (500 MHz, $CDCl_3$) δ 6.69–4.65 (m, 2H), 4.01 (dq, *J* = 7.87, 7.77, 7.77, 2.55 Hz, 1H), 2.58–2.52 (m, 1H), 2.10–2.02 (m, 1H), 1.80–1.73 (m, 1H), 1.69–1.54 (m, 3H), 1.52–1.46 (m, 1H), 1.41–1.34 (m, 5H), 1.32–1.26 (m, 3H), 1.25–1.18 (m, 1H), 0.91 (t, *J* = 7.05, 7.05 Hz, 3H), 0.19 (s, 9H). ^{13}C-NMR (125 MHz, $CDCl_3$) δ 102.73, 90.98. 74.87, 36.82, 34.04, 31.95, 28.64, 27.84, 23.42, 22.58, 14.08, 0.16. HRMS (ESI) calc. for $C_{14}H_{29}N_2OSi$ $[M + H]^+$ 269.2049, found 269.2059.

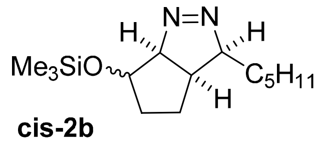

cis-2b

^{1}H-NMR (500 MHz, $CDCl_3$) δ 4.05 (td, *J* = 10.23, 6.42, 6.42 Hz, 1H), 3.85 (dd, *J* = 8.88, 7.05 Hz, 1H), 3.37 (t, *J* = 8.45, 8.45 Hz, 1H), 2.33–2.22 (m, 1H), 2.16–2.10 (m, 1H), 1.83–1.74 (m, 2H), 1.66–1.57 (m, 3H), 1.54–1.48 (m 1H), 1.32–1.22 (m, 7H), 0.89 (t, *J* = 6.34, 6.34 Hz, 3H), 0.12 (s, 9H). ^{13}C-NMR (125 MHz, $CDCl_3$) δ 75.96, 62.10, 51.91, 31.95, 31.73, 27.61, 26.36, 25.65, 22.49, 14.02, 0.01. HRMS (ESI) calc. for $C_{14}H_{29}N_2OSi$ $[M + H]^+$ 269.2049, found 269.2062.

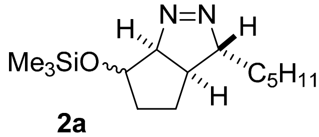

2a

^{1}H-NMR (500 MHz, $CDCl_3$) δ 4.90 (d, *J* = 7.44 Hz, 1H), 4.58 (bs, 1H), 4.30 (bs, 1H), 2.18 (t, *J* = 7.94, 7.94 Hz, 1H), 2.09–2.01 (m, 1H), 1.87–1.80 (m, 1H), 1.53–1.50 m, 1H), 1.43–1.39 (m, 2H), 1.33–1.26 (m, 6H), 1.18–1.12 (m, 1H), 0.90 (bt, *J* = 6.45, 6.45 Hz, 3H), 0.20 (s, 9H). ^{13}C-NMR (125 MHz, $CDCl_3$) δ 102.14, 97.46, 74.12, 38.80, 33.37, 32.62, 31.77, 30.43, 26.09, 22.53, 14.04, 0.16. HRMS (ESI) calc. for $C_{14}H_{29}N_2OSi$ $[M + H]^+$ 269.2049, found 269.2062.

1a

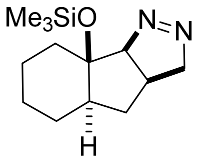

^{1}H-NMR (400 MHz, $CDCL_3$) δ 4.99 (dd, *J* = 10.1, 3.1 Hz, 1H), 4.55 (dd, *J* = 17.9, 10.5 Hz, 1H), 3.86 (ddd, *J* = 18.0, 6.4, 3.2 Hz, 1H), 2.57 (d, *J* = 13.1 Hz, 1H), 2.33–2.16 (m, 1H), 1.85 (ddd, *J* = 14.1, 8.6, 5.3 Hz, 1H), 1.73–1.51 (m, 4H), 1.47–1.37 (m, 3H), 1.28–1.05 (m, 3H), 0.08 (d, *J* = 3.0 Hz, 9H). ^{13}C-NMR (101 MHz, $CDCL_3$) δ 102.88, 83.26, 81.95, 77.31, 76.99, 76.67, 51.58, 36.05, 35.56, 32.94, 25.41, 24.55, 21.46, 2.64. HRMS (ESI) calc. for $C_{13}H_{25}N_2OSi$ $[M + H]^+$ 253.1736, found 253.1734.

1d

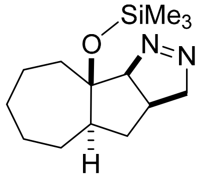

^{1}H-NMR (500 MHz, $CDCL_3$) δ 4.96 (dd, *J* = 10.1, 2.9 Hz, 1H), 4.57–4.42 (m, 1H), 3.97 (ddd, *J* = 17.9, 5.6, 3.2 Hz, 1H), 2.66 (ddd, *J* = 14.8, 5.8, 2.4 Hz, 1H), 2.32–2.19 (m, 1H), 1.94–1.79 (m, 3H), 1.76–1.44 (m, 10H), 1.39–1.30 (m, 1H), 1.14–1.04 (m, 1H), 0.13–0.06 (m, 9H). ^{13}C-NMR (101 MHz, $CDCL_3$) δ 104.68, 86.44, 82.77, 77.31, 76.99, 76.67, 51.19, 41.35, 38.51, 32.88, 27.60, 25.60, 24.19, 23.61, 2.67. HRMS (ESI) calc. for $C_{14}H_{27}N_2OSi$ $[M + H]^+$ 267.1893, found 267.1890.

1e

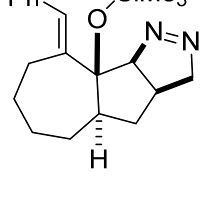

^{1}H-NMR (500 MHz, CDCl3) δ 7.62 (s, 1H), 7.37 (t, *J* = 7.6 Hz, 2H), 7.31–7.0 (m, 2H), 7.26–7.24 (m, 1H), 5.48 (dd, *J* = 10.3, 3.p Hz, 1H), 4.64(dd, *J* = 17.8, 10.3 Hz, 1H), 3.94 (ddd, *J* = 17.8, 6.6, 3.1 Hz, 1H), 2.65 (dd, *J* = 14.9, 7.8 Hz, 1H), 2.33–2.27 (m, 2H), 2.09–2.05 (m, 1H) 1.94 (ddd, *J* = 11.9, 8.7, 6.6 Hz, 1H), 1.81–1.72 (m, 4H), 1.51–1.42 (m, 2H), 1.26 (td, *J* = 12.2, 7.6 Hz, 1H), 0.05 (s, S-8 9Hz). ^{13}C-NMR (125 MHz, CDCl3) δ

145.37, 138.17, 130.58, 128.91, 128.20, 126.50, 103.13, 88.19, 83.30, 51.84, 37.35, 32.28, 27.83, 27.67, 27.29, 25.45, 2.84. HRMS (ESI) calc. for $C_{14}H_{31}N_2OSi$ $[M + H]^+$ 355.2206, found 355.2206.

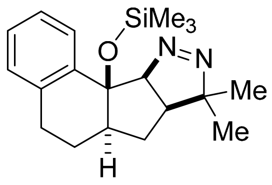

1c

^{1}H-NMR (400 MHz, CDCl3) δ 8.50–8.01 (m, 1H), 7.33–7.22 (m, 2H), 7.21–7.12 (m, 1H), 5.48 (d, *J* = 10.2 Hz, 1H), 3.02–2.80 (m, 2H), 2.15 (dd, *J* = 18.7, 8.8 Hz, 1H), 1.93 (pd, *J* = 12.6, 6.5 Hz, 2H), 1.82–1.60 (m, 2H), 1.55–1.44 (m, 1H), 1.38 (d, *J* = 5.6 Hz, 6H), −0.29 (s, 9H). ^{13}C-NMR (101 MHz, CDCl3) δ 138.78, 137.83, 129.27, 128.87, 128.26, 125.48, 102.74, 87.42, 80.54, 77.31, 76.99, 76.67, 49.02, 43.94, 29.54, 29.25, 29.03, 21.43, 20.62, 1.42. HRMS (ESI) calc. for $C_{19}H_{29}N_2OSi$ $[M + H]^+$ 329.2049, found 329.2049.

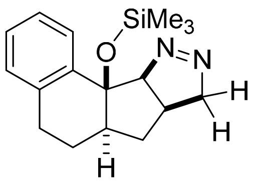

1b

^{1}H-NMR (400 MHz, CDCl3) δ 8.38–8.22 (m, 1H), 7.34–7.23 (m, 2H), 7.15 (dd, *J* = 5.0, 3.8 Hz, 1H), 5.47 (dd, *J* = 10.0, 2.9 Hz, 1H), 4.70 (dd, *J* = 17.7, 10.5 Hz, 1H), 3.91 (ddd, *J* = 17.7, 7.1, 3.0 Hz, 1H), 3.00–2.79 (m, 2H), 2.52–2.29 (m, 1H), 2.14–1.82 (m, 3H), 1.79–1.65 (m, 1H), 1.43–1.17 (m, 2H), 0.32 (s, 9H). ^{13}C-NMR (101 MHz, CDCl3) δ 138.21, 137.78, 129.48, 128.88, 128.38, 125.55, 101.29, 83.00, 81.48, 77.31, 76.99, 76.67, 49.15, 34.59, 34.15, 29.07, 20.98, 1.35. HRMS (ESI) calc. for $C_{17}H_{25}N_2OSi$ $[M + H]^+$ 301.1736, found 301.1737.

4. Computational Data

4.1. Table of Energy Values (in a.u.)

Species	Enthalpy	Free Energy
**IN-1b**	−983.704537	−983.777262
***syn*-TS-1b**	−983.679348	−983.746287
***anti*-TS-1b**	−983.67826	−983.745288
***syn*-1b**	−983.74574	−983.810444
***anti*-1b**	−983.749865	−983.815234
**IN-1b′**	−983.705843	−983.779457
***syn*-TS-1b′**	−983.679466	−983.745844
***anti*-TS-1b′**	−983.673821	−983.740303
***syn*-1b′**	−983.747524	−983.812473
***anti*-1b′**	−983.747361	−983.813072
**IN-2a**	−867.043546	−867.114206
***syn*-TS-2a**	−867.012163	−867.077092
***anti*-TS-2a**	−867.014639	−867.079784
***syn*-2a**	−867.081985	−867.144665
***anti*-2a**	−867.085089	−867.148929
**IN-2b**	−827.7535	−827.820651
***syn*-TS-2b**	−827.725581	−827.786484
***anti*-TS-2b**	−827.727787	−827.789079

***syn*-2b**	−827.793632	−827.852806
***anti*-2b**	−827.797055	−827.856769
**IN-2c**	−867.040993	−867.111917
***syn*-TS-2c**	−867.009754	−867.073989
***anti*-TS-2c**	−867.011746	−867.076989
***syn*-2c**	−867.079217	−867.142494
***anti*-2c**	−867.083341	−867.145937
**IN-3a**	−867.03829	−867.106675
***syn*-TS-3a**	−867.010656	−867.073362
***anti*-TS-3a**	−867.01093	−867.072946
***syn*-3a**	−867.079024	−867.140659
***anti*-3a**	−867.078842	−867.140627

4.2. Cartesian Coordinates for All Species

IN-1b

C	1.43846400	3.44680200	-0.58971600
C	2.65870900	4.34459500	-0.78524000
C	2.21124700	5.75903800	-1.13882600
C	1.27944200	6.37116600	-0.07573200
C	0.06715600	5.45373500	0.19087200
C	0.53371300	4.02450700	0.49545000
H	0.88402300	3.37121100	-1.53363300
H	3.24402700	4.37349900	0.13895700
H	1.67450100	5.74903400	-2.09430300
H	-0.34669600	3.39445000	0.64724400
H	3.07057000	6.42781700	-1.25304700
H	3.30802600	3.95576100	-1.57406400
H	1.74548800	2.43318400	-0.31918300
H	1.08458500	4.04728500	1.44262900
H	-0.53344400	5.43163300	-0.72983900
C	0.86638400	7.74197200	-0.58249700
H	1.59397400	8.45378500	-0.94727000
N	-0.36814600	8.04080300	-0.82005400
N	-1.45930100	8.28234900	-1.00250600
O	2.02248200	6.45981000	1.13495300
C	-0.80903600	5.96176400	1.35484400
H	-0.31557900	5.70699000	2.29747100
H	-0.89640700	7.05193400	1.33570000
C	-2.18757200	5.37026300	1.31640000
H	-2.79946700	5.65253100	0.46009300
C	-2.68873500	4.54205300	2.22589200
H	-2.10642900	4.23921900	3.09108500
H	-3.69284500	4.14381300	2.13986700
Si	2.44166200	7.75217800	2.10692900
C	0.95343100	8.65337500	2.80234000
H	1.29226700	9.47088800	3.44654100
H	0.33549500	9.08830400	2.01252100
H	0.32361100	7.99499500	3.40524000
C	3.41465700	6.95148400	3.48465600
H	3.75256200	7.69207300	4.21437600
H	2.80360000	6.21308300	4.00925000
H	4.29466300	6.43831800	3.08983700
C	3.52619900	8.97659900	1.18139300
H	4.09261300	9.58415500	1.89346900
H	4.24863200	8.46151700	0.54238900
H	2.94403300	9.66209900	0.56152600

syn-TS-1b

C	1.03811700	3.55244900	0.49685600
C	2.43539700	3.94608600	-0.00812000
C	2.38417100	5.11586600	-1.00688600
C	1.62600300	6.33818300	-0.44463800
C	0.20543900	5.89268200	0.02284900
C	0.27705500	4.76556500	1.05910300
H	0.45959800	3.11888400	-0.33309200
H	3.06203700	4.22083700	0.84840300
H	1.86534700	4.78985200	-1.91893300
H	-0.74057900	4.47175100	1.34960600
H	3.39386400	5.41249700	-1.31269600
H	2.92184200	3.08642000	-0.48717900
H	1.12385100	2.76962700	1.26166700
H	0.77435800	5.13423500	1.96616300
H	-0.29140100	5.48672400	-0.87377800
O	2.28178100	6.92501300	0.66833500
C	1.31655900	7.40133800	-1.50596600
H	1.09614600	7.03668600	-2.50843700
N	1.84314290	8.61866745	-1.72418237
N	1.61218461	9.75158240	-1.56414267
C	-0.57095100	7.14824400	0.44463100
H	-1.60876800	6.87147100	0.67916600
H	-0.13397000	7.56750000	1.35864800
C	-0.55258400	8.18881700	-0.66398500
H	-1.12670000	7.92303900	-1.55317000
C	-0.31061300	9.53307100	-0.44678300
H	-0.61963200	10.28616600	-1.16555000
H	-0.01389000	9.89792700	0.53354200
Si	3.81657000	7.38866900	1.18843300
C	5.10657000	7.44796200	-0.19103000
H	6.03266300	7.88193000	0.21028400
H	5.35489600	6.45196800	-0.57666200
H	4.78673000	8.07135200	-1.03383800
C	3.59570400	9.09752600	1.95282800
H	4.53466900	9.43977700	2.40768500
H	3.29108300	9.83925200	1.20564800
H	2.83149300	9.07781100	2.74002200
C	4.38814600	6.19636200	2.53787300
H	5.28223900	6.59705200	3.03444400
H	3.61363600	6.06602100	3.30412900
H	4.64537200	5.20590300	2.14463000

anti-TS-1b

C	0.78452600	3.41984400	-0.07913800
C	2.29946000	3.63037000	-0.15123000
C	2.64160700	4.94453400	-0.85712400
C	1.95871700	6.12488700	-0.15483200
C	0.43500600	5.91495100	-0.10529700
C	0.09121700	4.60529500	0.59911000
H	0.38882700	3.30426700	-1.09585400
H	2.71027800	3.65470600	0.86355200
H	2.29426100	4.91440900	-1.89591500
H	-0.99348000	4.46336800	0.60346600
H	3.72201400	5.11229700	-0.88050800
H	2.77668100	2.79712700	-0.67365000
H	0.55875900	2.49339800	0.45525300
H	0.41662200	4.67580300	1.64252500
H	0.08811100	5.84993100	-1.14694200
O	2.40200100	6.20683300	1.18539700
C	2.19060300	7.47628600	-0.82620600
H	3.07646800	8.04141000	-0.55035000
N	1.12185000	7.94844500	-2.88268300
N	1.90820500	7.57294900	-2.13340900
C	-0.17919800	7.16495600	0.52959200
H	-1.27012700	7.10011600	0.48280000
H	0.10375600	7.19001800	1.58494800
C	0.29974500	8.42590800	-0.17681700
H	0.81951600	9.16515100	0.42465200
C	-0.28214700	8.83305100	-1.35586700
H	-1.09960300	8.26511500	-1.78757200
H	-0.15607000	9.84231700	-1.72704200
Si	3.74080700	6.83665800	1.95315000
C	3.81725600	5.92332600	3.57745000
H	3.94736200	4.85115700	3.41136900
H	4.64865600	6.27424500	4.19416000
H	2.89289100	6.06566700	4.14230100

C	5.30528200	6.54570200	0.96043000
H	6.16271800	6.98715000	1.47686200
H	5.50107200	5.47637200	0.84485000
H	5.25880600	6.98852400	-0.03806000
C	3.48254400	8.66866000	2.25843500
H	2.50640400	8.83242000	2.72395300
H	4.24545100	9.05654200	2.93949000
H	3.51986400	9.26769100	1.34535200

syn-1b

C	1.63031100	3.69600200	0.01311800
C	2.86338900	4.48116800	-0.45503900
C	2.47826800	5.73867000	-1.24414500
C	1.51699000	6.59273200	-0.42827900
C	0.26887600	5.78501800	-0.03206600
C	0.64981800	4.57349600	0.80467100
H	1.11281200	3.28971100	-0.86506400
H	3.45455700	4.77058000	0.41863000
H	1.96720100	5.44882800	-2.16948200
H	-0.23958500	3.99868700	1.07915700
H	3.35820900	6.31851400	-1.53228800
H	3.50255300	3.84192400	-1.06969900
H	1.94404100	2.84026500	0.61648800
H	1.11921800	4.91889800	1.73180600
H	-0.18875000	5.43449800	-0.96873100
O	2.08357100	7.05060300	0.78615500
C	0.86148200	7.78940000	-1.15169800
H	0.68690800	7.54472300	-2.20125800
N	1.69802000	8.99812400	-1.08809600
N	1.25394000	9.83631600	-0.30591400
C	-0.64142400	6.85674800	0.57761700
H	-1.68756900	6.54803200	0.60926000
H	-0.31647000	7.06825900	1.59927800
C	-0.41666200	8.09119800	-0.33144400
H	-1.27000700	8.24925000	-0.99176400
C	-0.01509800	9.40473800	0.34286800
H	-0.73287300	10.21866700	0.23320700
H	0.21252600	9.29132700	1.40730300
Si	3.53411300	7.64369400	1.37009400
C	4.18780700	6.40703200	2.62331200
H	4.60527400	5.50831500	2.16495500
H	4.97867000	6.86772100	3.22278500
H	3.39113700	6.09997800	3.30651500
C	3.15285200	9.22928500	2.28561100
H	4.04057600	9.56973100	2.82699400
H	2.83794900	10.02550600	1.60825700
H	2.36125600	9.06606300	3.02280000
C	4.80256500	7.93967600	0.02515000
H	5.63703800	8.51263900	0.44079700
H	5.20885000	7.00547900	-0.37045700
H	4.37120400	8.51746800	-0.79613800

anti-1b

C	0.89095100	3.35715000	-0.62757700
C	2.39560700	3.63240200	-0.75478600
C	2.68067000	5.09657000	-1.11509200
C	2.01755900	6.00164600	-0.08392500
C	0.50373100	5.74552500	-0.02305900
C	0.21081200	4.30508100	0.37095200
H	0.42184800	3.48266200	-1.61141300
H	2.88524800	3.39740800	0.19584900
H	2.27226600	5.33350600	-2.10174700
H	-0.86774000	4.12456400	0.39535800
H	3.75615900	5.28945500	-1.15832700
H	2.83042500	2.97560800	-1.51254200
H	0.72801400	2.31767200	-0.33104600
H	0.60074300	4.12951500	1.37836800
H	0.11634100	5.91324100	-1.03860300
O	2.52432400	5.71586600	1.21464100
C	2.07123300	7.52316200	-0.31756500
H	3.03750600	7.94511500	-0.02957200
N	0.90790300	8.56544200	-1.96044900
N	1.88772300	7.85482700	-1.75290900
C	0.01370300	6.87796700	0.88053800
H	-1.06255100	7.04944900	0.80687900
H	0.25046800	6.63677600	1.91924500
C	0.83437600	8.10579500	0.41468400
H	1.10977300	8.75139500	1.24723300
C	0.17580400	8.90655300	-0.71487000
H	-0.87778200	8.66595100	-0.87695800
H	0.25118100	9.99029400	-0.59471400
Si	3.92349300	6.14177800	2.01158800
C	3.71213700	7.82770700	2.81142200
H	4.51265700	8.00946800	3.53442300
H	3.73010900	8.64768400	2.08928100
H	2.76201500	7.87334700	3.35118400
C	4.12948300	4.83932000	3.33283900
H	4.24756400	3.84957500	2.88496200
H	5.00791600	5.03910800	3.95224000
H	3.25407200	4.81020200	3.98610200
C	5.40948700	6.15456200	0.86699300
H	6.30538500	6.43501100	1.42890200
H	5.58240100	5.16463200	0.43652200
H	5.29988400	6.86575300	0.04396900

IN-1b'

C	1.56744100	3.27029600	-0.20477000
C	2.83555000	4.11900600	-0.09750900
C	2.55227600	5.56837400	-0.49611200
C	1.42042400	6.20061800	0.32987500
C	0.14495100	5.33139400	0.19289900
C	0.42369000	3.88241500	0.60432300
H	1.26679200	3.20889300	-1.25793000
H	3.22122800	4.08077600	0.92730000
H	2.24130100	5.58291300	-1.54918900
H	-0.48999700	3.29479400	0.48276400
H	3.44399600	6.19426100	-0.40380500
H	3.62361700	3.71283700	-0.73657700
H	1.76456900	2.24798400	0.12813900
H	0.66789600	3.85750300	1.67434000
H	-0.10440300	5.33252500	-0.87753600
C	-1.04149800	5.92799800	0.96247300
H	-0.89087700	5.78475200	2.03762600
H	-1.06732800	7.00741900	0.77779400
C	-2.34663500	5.31886600	0.54366200
H	-2.63287000	5.47538600	-0.49624800
C	-3.14770400	4.60830600	1.32995600
H	-2.89724500	4.42723000	2.37086700
H	-4.07891900	4.19145100	0.96443500
C	1.85228000	6.32924800	1.78359600
H	2.30513000	5.54325700	2.36507400
N	1.63272700	7.44147200	2.40303800
N	1.43786600	8.42916000	2.91813100
O	1.14751500	7.51755200	-0.10026300
Si	1.02260200	8.25378600	-1.59338900
C	-0.09690500	7.28677000	-2.74929600
H	-0.24991800	7.85368800	-3.67252900
H	0.32142100	6.31575800	-3.02697700
H	-1.07810400	7.11872100	-2.29640800
C	2.70971400	8.47334400	-2.38017200
H	3.12936100	7.53107100	-2.73929000
H	2.64027200	9.15355500	-3.23416900
H	3.41323500	8.90721600	-1.66489700
C	0.26461100	9.91241300	-1.20576400
H	0.89434400	10.47268000	-0.51085700
H	0.14026200	10.51184700	-2.11144000
H	-0.71723900	9.78956400	-0.74199300

syn-TS-1b′

C	1.10632300	3.27869900	-0.35129000
C	2.31957500	3.59488700	0.52380600
C	2.80719800	5.02500600	0.28418800
C	1.70567100	6.07708800	0.47433400
C	0.46261900	5.73060600	-0.37544800
C	-0.01110500	4.29589400	-0.11786500
H	1.40688300	3.31024000	-1.40544500
H	2.05775700	3.45444800	1.57868700
H	3.15447900	5.11176100	-0.75201600
H	-0.86358100	4.08458300	-0.76991600
H	3.65464200	5.26046100	0.93594100
H	3.13406600	2.89652900	0.31594400
H	0.74804900	2.26540200	-0.15238000
H	-0.38027500	4.20248800	0.91089800
H	0.76785100	5.81285200	-1.42609800
C	-0.60701400	6.77658000	-0.04887100
H	-0.30433600	7.73819300	-0.46840200
H	-1.55357400	6.49335400	-0.52221800
C	-0.80581100	6.93821400	1.44738200
C	-0.84249100	8.17762800	2.04605900
O	2.12805300	7.35726300	0.06726300
C	1.18125400	6.17038100	1.91043000
H	1.10114000	5.26216300	2.49857500
N	1.55914100	7.23514100	2.62464800
N	1.22533800	8.28087000	2.96232800
H	-1.32248400	6.12503800	1.95191600
H	-1.31977200	8.32441600	3.00662000
H	-0.64492200	9.07158600	1.46451700
Si	3.59859300	8.15951000	0.05931500
C	4.71958300	7.45420700	-1.27050600
H	4.17382800	7.30268500	-2.20533700
H	5.53931700	8.15016400	-1.47219800
H	5.16256200	6.50021500	-0.97461800
C	3.12479500	9.91740700	-0.34336300
H	4.00226300	10.56924900	-0.35914200

H	2.63538600	9.98103600	-1.31789200
H	2.43087400	10.29977700	0.40956100
C	4.48789600	8.09373400	1.70841200
H	3.94710800	8.64831100	2.47806500
H	4.63606100	7.07397700	2.07190500
H	5.47697900	8.54875000	1.59423700

anti-TS-1b′

C	1.14585800	2.91043800	-0.49137100
C	2.54167500	3.30302500	-0.00790500
C	2.89241500	4.71698400	-0.47241400
C	1.85925700	5.77588300	-0.06069500
C	0.42669900	5.34696500	-0.42976000
C	0.10055900	3.91039900	0.00213600
H	1.13832300	2.88917300	-1.58803300
H	2.58858200	3.23760000	1.08331900
H	2.92929300	4.73060000	-1.56812200
H	-0.88639800	3.65391500	-0.39443400
H	3.88148600	5.01316500	-0.10655200
H	3.29044900	2.60585500	-0.39271900
H	0.89586700	1.90266200	-0.15057800
H	0.01665300	3.84508500	1.09057000
H	0.37401900	5.38774900	-1.52480200
O	2.08321400	6.99581500	-0.75068000
C	1.87975800	6.15609900	1.42242900
H	2.52817800	6.98480200	1.68814400
N	1.82849600	5.22839000	2.38603100
N	1.15789100	4.75285900	3.18827200
C	-0.54389100	6.37849000	0.16927900
H	-0.51243100	7.28117300	-0.44276900
H	-1.56253200	5.98305900	0.11470900
C	-0.20934600	6.75068800	1.61056800
H	0.06937400	7.78696700	1.77600600
C	-0.68649400	6.04499500	2.69077500
H	-1.29795400	5.16018600	2.55217000
H	-0.70075200	6.48202800	3.68118800
Si	3.47063800	7.83046800	-1.16664600
C	4.59238900	8.08912600	0.31619100
H	4.11016600	8.69193000	1.09031500
H	4.91274300	7.14632200	0.76803400
H	5.49381700	8.62310900	0.00078200
C	2.83347800	9.46611600	-1.79834200
H	3.65619500	10.12007000	-2.09895100
H	2.18454000	9.31813000	-2.66472600
H	2.25323000	9.98032500	-1.02882100
C	4.43283700	6.95603000	-2.51905100
H	4.96919000	6.07748100	-2.15419000
H	3.76987900	6.63900300	-3.32824700
H	5.17088600	7.64238100	-2.94513900

syn-1b′

C	0.95494800	3.02980900	0.06375400
C	2.31013300	3.71295700	0.25078500
C	2.31940800	5.09620100	-0.40564100
C	1.14585800	5.97238200	0.02813200
C	-0.22641500	5.27095900	0.00495600
C	-0.16437100	3.88477200	0.65614300
H	0.76909800	2.88160300	-1.00753400
H	2.52785000	3.79604200	1.32122200
H	2.25965100	4.96696400	-1.49036900
H	-1.13513500	3.39456600	0.53747800
H	3.24688700	5.63595200	-0.19366100
H	3.10888500	3.10376300	-0.17931700
H	0.95988500	2.03906900	0.52545400
H	-0.00358600	3.99468900	1.73645900
H	-0.55612700	5.14913400	-1.03445000
C	-1.12893200	6.24057800	0.79104300
H	-1.52766800	7.01447500	0.13388500
H	-1.97973200	5.71820500	1.23425600
C	-0.20888100	6.87032200	1.86610900
C	-0.06032200	8.39593000	1.85121100
O	1.09519100	7.15519500	-0.75015800
C	1.22652600	6.44920300	1.50067800
H	1.65111400	5.67282200	2.13765900
N	2.06844200	7.65454300	1.63183000
N	1.40165400	8.67085200	1.80667800
H	-0.47676800	6.52772200	2.86608400
H	-0.46863400	8.90603900	2.72445600
H	-0.48488700	8.85727900	0.95425000
Si	0.64824900	7.52651700	-2.31165900
C	1.54599100	9.11638100	-2.68718800
H	2.62699500	8.96043300	-2.67810100
H	1.26366600	9.51404400	-3.66553000
H	1.31559800	9.87085200	-1.93089500
C	1.12281900	6.18915600	-3.54038100
H	0.78834300	6.48740200	-4.53867200
H	2.20462900	6.04292900	-3.58425800

H	0.65635800	5.22634300	-3.31354200
C	-1.20528900	7.80526000	-2.43186200
H	-1.53837300	8.57695600	-1.73259900
H	-1.45872500	8.14551600	-3.44050100
H	-1.78036800	6.89665700	-2.23609700

anti-1b′

C	1.09840400	2.93253200	-0.61796800
C	2.46884500	3.28604500	-0.04048900
C	2.86134500	4.72029900	-0.40537000
C	1.79876700	5.77737000	-0.09438100
C	0.36802300	5.35321300	-0.47566300
C	0.03996800	3.90281800	-0.09615400
H	1.13365000	2.98698300	-1.71332200
H	2.45117000	3.16535100	1.04476500
H	3.02718300	4.76682000	-1.48755800
H	-0.94635200	3.65767800	-0.50174500
H	3.80408500	4.99526700	0.08207100
H	3.23158900	2.60465000	-0.42679100
H	0.83200800	1.90511000	-0.35694600
H	-0.03621900	3.78883700	0.99048700
H	0.27622700	5.45556800	-1.56140000
O	2.04366300	6.97232600	-0.83499900
C	1.67304800	6.21834600	1.38652200
H	2.43891400	6.96402100	1.60446400
N	1.88617700	5.12752200	2.36830000
N	0.92136700	4.94939500	3.10783700
C	-0.50947000	6.38141200	0.25540600
H	-0.57720000	7.29149900	-0.34156200
H	-1.52473400	6.01010100	0.41999900
C	0.21856600	6.69449400	1.58334900
H	0.16720300	7.75736600	1.81289900
C	-0.20852100	5.86225800	2.79925800
H	-1.09178300	5.24063300	2.62751100
H	-0.39370500	6.45186400	3.70010800
Si	3.44159600	7.82072300	-1.17295200
C	4.44463800	8.16924100	0.37635700
H	3.89035400	8.78412500	1.09024100
H	4.75864100	7.25374000	0.88527500
H	5.35087900	8.71838100	0.10350200
C	2.82745900	9.41786800	-1.91888100
H	3.66019100	10.07566700	-2.18154400
H	2.25135700	9.22303900	-2.82646600
H	2.18071200	9.95117600	-1.21837600
C	4.53152100	6.92220600	-2.40693200
H	5.04880300	6.06951300	-1.96213800
H	3.94830200	6.56159600	-3.25826200
H	5.29156600	7.60848800	-2.79247700

IN-2a

C	0.98292900	4.03619300	0.43968400
C	1.10347600	2.76188500	0.08016600
H	1.66611600	2.52099400	-0.82222200
C	0.53286200	1.59500700	0.83180400
H	-0.03165500	1.95734100	1.69794000
H	-0.17589700	1.05776100	0.19090900
C	1.62226800	0.62289700	1.28828000
H	2.19398200	0.26007900	0.42605400
H	2.33254200	1.13323000	1.94367000
C	1.07833800	-0.58754600	2.04324600
H	0.48249200	-0.23998100	2.89831000
C	0.13685100	-1.43393400	1.21500500
H	-0.89279100	-1.64489400	1.45618500
N	0.59246300	-1.96627900	0.12427600
N	1.04257100	-2.42625800	-0.80443300
O	2.20024000	-1.31727600	2.50848000
Si	2.12655100	-2.76801200	3.34696800
C	3.77842200	-2.90595300	4.20209800
H	3.85848600	-3.84551200	4.75508400
H	4.59082700	-2.87424400	3.47220900
H	3.92645500	-2.08279800	4.90433200
C	0.72028400	-2.68701700	4.58565700
H	0.83059200	-1.82724500	5.25193200
H	-0.25163500	-2.61152100	4.09022600
H	0.70270900	-3.58740200	5.20597600
C	1.88329800	-4.21118600	2.17794100
H	0.86842800	-4.24610300	1.77485000
H	2.57803600	-4.14863100	1.33623500
H	2.07098100	-5.15676000	2.69474100
H	0.42194200	4.27204200	1.34325600
C	1.56110600	5.19743700	-0.31222600
H	2.11423400	4.86096600	-1.19078200
H	0.77392700	5.88036200	-0.64392000
H	2.24021900	5.77577500	0.32029900

syn-TS-2a

C	0.62583700	0.35825200	-1.80742300
C	0.92844700	0.89957800	-0.57709500
H	0.32737000	1.74548000	-0.24229600
C	2.29566400	0.77976100	0.06599300
H	2.88029400	0.02547700	-0.46642400
H	2.83547400	1.72809400	-0.01810700
C	2.17378100	0.37984400	1.53680600
H	3.14052200	0.16426200	1.99918900
H	1.69937000	1.18442900	2.10995400
C	1.28205100	-0.86047400	1.59761500
H	1.06845300	-1.12722200	2.64171900
C	0.00254100	-0.46424100	0.87201000
H	-0.76327100	0.06651400	1.42879300
N	-0.46710800	-1.31156000	-0.04482800
N	-0.46071600	-1.53117700	-1.17462900
O	1.92006100	-1.91832900	0.92185100
Si	1.84451900	-3.52039100	1.43453700
C	2.67740900	-4.48890100	0.07964400
H	2.68131100	-5.55858300	0.30556700
H	2.16035400	-4.34978300	-0.87306000
H	3.71469500	-4.17090200	-0.05091300
C	2.76146300	-3.67148600	3.05967800
H	3.79155400	-3.31828100	2.96502500
H	2.28113000	-3.09495400	3.85531900
H	2.79558100	-4.71373800	3.38993100
C	0.05806000	-4.02681600	1.66367900
H	-0.46266600	-3.35611700	2.35377200
H	-0.48772400	-4.01849300	0.71726100
H	-0.00285300	-5.03662600	2.07983300
H	1.38600600	-0.24135500	-2.30308300
C	-0.49748000	0.87609500	-2.66695400
H	-0.15791300	1.67532800	-3.33490400
H	-0.92590700	0.08960600	-3.29230500
H	-1.30199200	1.28644600	-2.04979500

anti-TS-2a

C	1.36095000	0.22130200	-1.81572200
C	0.81641200	0.57802600	-0.60243700
H	-0.27046000	0.62069600	-0.53190100
C	1.56266500	1.39968400	0.43118100
H	2.63194100	1.39553000	0.19196100
H	1.23492700	2.44311300	0.39354600
C	1.34468100	0.82868100	1.82961300
H	1.94644000	1.32785300	2.59263200
H	0.29274600	0.92220100	2.12177900
C	1.69847300	-0.65593200	1.77488300
C	0.90497700	-1.23220900	0.61911000
H	-0.09895100	-1.59196600	0.82741900
N	1.56700500	-1.95906600	-0.29151100
N	2.04463000	-1.89966400	-1.33625400
H	2.76637500	-0.75156500	1.53473600
O	1.42436000	-1.26466700	3.01761900
Si	1.83518200	-2.85751200	3.38556800
C	0.57264500	-4.04199400	2.66969300
H	-0.44454400	-3.74434000	2.93860600
H	0.63199600	-4.09850900	1.57991200
H	0.73580300	-5.05028700	3.06238200
C	3.53194000	-3.22277500	2.68424200
H	4.27433600	-2.49930700	3.03251100
H	3.86569000	-4.21563200	2.99894000
H	3.53565100	-3.21174900	1.59041100
C	1.82080300	-2.93332800	5.24821900
H	2.07197300	-3.93734500	5.60064300
H	2.54385100	-2.23570000	5.67772700
H	0.83399900	-2.67998800	5.64396400
H	2.39383000	0.49605500	-2.01740600
C	0.51574900	-0.15956400	-3.00238200
H	0.26704700	0.71328800	-3.61559600
H	1.02525700	-0.87749900	-3.64878400
H	-0.42650100	-0.61109600	-2.67892700

syn-2a

C	0.62903100	-1.37876300	-1.28975000
C	0.79846900	-0.04567900	-0.54844100
H	0.08370800	0.68390500	-0.93590300
C	2.22780000	0.54446900	-0.47731000
H	2.93957400	-0.13026700	-0.95646900
H	2.30132900	1.51293800	-0.97256300
C	2.53964700	0.63567100	1.02889600
H	3.60580600	0.60278000	1.25795900
H	2.11822900	1.55174100	1.45497600
C	1.80710700	-0.57457800	1.60112400
C	0.43869900	-0.46733400	0.88792800
H	-0.21689100	0.21768200	1.42818400
N	-0.21992300	-1.78144300	0.78389600
N	-0.09839000	-2.27093600	-0.33706700
H	1.71429800	-0.55843100	2.69277200
O	2.46902800	-1.73502500	1.14956300
Si	2.74478600	-3.13424600	2.03460600
C	1.23051700	-3.63139300	3.01229900
H	1.46629700	-4.49365600	3.64339000
H	0.88631900	-2.82703300	3.66824600
H	0.40134500	-3.89351200	2.35303600
C	4.17022900	-2.80585900	3.20727900
H	3.92344200	-2.00965400	3.91573400
H	4.41831900	-3.69791800	3.78883900
H	5.06434500	-2.50123700	2.65777500
C	3.18310100	-4.40980600	0.74946300
H	3.39093600	-5.38149000	1.20468200
H	2.34932200	-4.53264800	0.05326200
H	4.06395500	-4.10676100	0.17862600
H	1.59806100	-1.86828900	-1.43886700
C	-0.13513000	-1.31437800	-2.60171300
H	0.40469500	-0.69693000	-3.32287500
H	-0.26402200	-2.31220600	-3.02329400
H	-1.12434200	-0.87687300	-2.44476200

anti-2a

C	0.43760800	0.12081600	-1.91131900
H	1.22501900	0.47985800	-2.57970500
C	0.76489700	0.32800200	-0.43472000
H	0.18965200	1.14722800	-0.00005400
C	2.25994900	0.46851400	-0.09633100
H	2.85294800	-0.08833700	-0.83114900
H	2.60509000	1.50323000	-0.10918500
C	2.37830500	-0.19622200	1.27633400
H	3.40672800	-0.43408000	1.55920700
H	1.95003700	0.44913000	2.05077200
C	1.49256000	-1.44558900	1.15339800
C	0.38105200	-1.02917900	0.17242800
H	-0.58509800	-1.07584400	0.67880800
N	0.31769100	-1.95500300	-0.99338100
N	0.37838900	-1.36644100	-2.06938300
H	2.06328800	-2.26344700	0.69180700
O	0.93893300	-1.87825200	2.37354000
Si	1.80949800	-2.57571200	3.62103400
C	0.55464600	-3.49485200	4.64741300
H	-0.22043100	-2.81350700	5.00602100
H	0.06918900	-4.27700700	4.05979600
H	1.02218900	-3.96176600	5.51809800
C	3.10226000	-3.72830100	2.90426200
H	3.86849500	-3.18413000	2.34499000
H	3.60946600	-4.27306200	3.70536200
H	2.65113200	-4.46308700	2.23259200
C	2.64684200	-1.24877500	4.64763300
H	3.13879400	-1.69161800	5.51841200
H	3.40608700	-0.71027800	4.07558800
H	1.91739900	-0.52027200	5.01094900
C	-0.91134400	0.68258400	-2.34772800
H	-0.89397600	1.77349200	-2.32474900
H	-1.15308500	0.35594100	-3.35995400
H	-1.70111500	0.33369700	-1.67693100

IN-2b

C	0.93953600	4.04175200	0.48804100
H	0.37505600	4.30201100	1.37826100
H	1.38228000	4.85643900	-0.07241300
C	1.07744400	2.77775500	0.10510300
H	1.65287500	2.55413100	-0.79194400
C	0.50939300	1.59347100	0.83015900
H	-0.07865900	1.93621700	1.68834200
H	-0.17515400	1.05376500	0.16557000
C	1.60723300	0.63539600	1.29630600
H	2.20033100	0.29002400	0.44151500
H	2.29644600	1.15111300	1.96969000
C	1.06841300	-0.59008800	2.03067600
H	0.44734700	-0.25865800	2.87410200
C	0.16166000	-1.44801800	1.17596300
H	-0.87161000	-1.67230600	1.38774400
N	0.65661100	-1.97670200	0.10050100
N	1.14043100	-2.43259900	-0.81304000
O	2.19277800	-1.30183500	2.51534600
Si	2.12495100	-2.75217600	3.35600400
C	3.75688800	-2.85739100	4.25236000
H	3.83928100	-3.79297000	4.81168900
H	4.58661600	-2.81484000	3.54283200
H	3.87291700	-2.02860300	4.95401500
C	0.68481700	-2.69494500	4.55691100
H	0.75243500	-1.82547500	5.21626300
H	-0.27471000	-2.65254800	4.03409900
H	0.67500400	-3.58885900	5.18669700
C	1.93706600	-4.20106200	2.18414000
H	0.93322900	-4.25602600	1.75626200
H	2.65114500	-4.12700300	1.35970700
H	2.12947100	-5.14214100	2.70738000

syn-TS-2b

C	0.69087100	0.27725900	-1.78909700
H	-0.11517200	0.61850200	-2.42569500
H	1.43672100	-0.35816300	-2.25375500
C	0.95457400	0.88453100	-0.58139300
H	0.35525500	1.75099700	-0.30871200
C	2.31119900	0.79235700	0.09200100
H	2.90764000	0.02906700	-0.41245300
H	2.84103800	1.74426400	-0.00465300
C	2.16726600	0.41715700	1.56803500
H	3.12674800	0.20209000	2.04468500
H	1.68547100	1.23042300	2.12307100
C	1.27019800	-0.82005000	1.62432500
H	1.05176300	-1.09241700	2.66712700
C	0.00008700	-0.41388200	0.88745400
H	-0.75809000	0.13598900	1.43629100
N	-0.48859000	-1.28240300	-0.00476400
N	-0.49459500	-1.53531500	-1.12530100
O	1.90803100	-1.87220000	0.94611000
Si	1.83187200	-3.48548600	1.40669200
C	2.63982300	-4.41319000	0.01000600
H	2.64539500	-5.48964700	0.19839500
H	2.10261000	-4.23355300	-0.92424100
H	3.67318300	-4.08617800	-0.12552900
C	2.77119300	-3.68964200	3.01543500
H	3.80084400	-3.33845500	2.91349400
H	2.30253100	-3.12968200	3.82961800
H	2.80169200	-4.74018800	3.31766200
C	0.04847300	-3.99708800	1.65380500
H	-0.46252400	-3.32952400	2.35407900
H	-0.50629600	-3.98039200	0.71307700
H	-0.00474800	-5.00928000	2.06458100

anti-TS-2b

C	1.40937100	0.23122600	-1.79581600
H	0.81569200	-0.05509000	-2.65448900
H	2.44561200	0.48379800	-1.99238900
C	0.81752700	0.58929300	-0.60554500
H	-0.26830700	0.64363600	-0.57315500
C	1.53742200	1.42118000	0.44158200
H	2.60766200	1.43629800	0.20769700
H	1.18933700	2.45712600	0.40019000
C	1.32299600	0.83989600	1.83658800
H	1.92112200	1.33383300	2.60518500
H	0.27109300	0.92002300	2.13204500
C	1.68451200	-0.64219600	1.76618900
C	0.87989500	-1.20467900	0.61163700
H	-0.12487600	-1.55337000	0.83495400
N	1.52649000	-1.95495000	-0.29569400
N	1.99585300	-1.92485600	-1.34307800
H	2.74996600	-0.73083100	1.50888200
O	1.42639500	-1.26154300	3.00347400
Si	1.83727000	-2.84863300	3.37115200
C	0.56650300	-4.03557900	2.67107100
H	-0.44482800	-3.74070400	2.96299800
H	0.61005200	-4.08074900	1.58019300
H	0.74124400	-5.04576000	3.05290700
C	3.52419700	-3.22447000	2.64840400
H	4.27534100	-2.51017700	2.99611100
H	3.85126100	-4.22329300	2.95008900
H	3.51043800	-3.20296500	1.55488300
C	1.84508600	-2.92463900	5.23360700
H	2.09232400	-3.92953000	5.58543200
H	2.57649200	-2.22793700	5.64896000
H	0.86361300	-2.65920300	5.63335600

syn-2b

C	0.60645500	-1.40988600	-1.29821000
H	0.00272200	-1.35796100	-2.20510500
H	1.54674000	-1.91488200	-1.53789400
C	0.81172100	-0.07323900	-0.57765100
H	0.11184300	0.67277800	-0.95544500
C	2.25471400	0.48473800	-0.52191300
H	2.94952200	-0.21842200	-0.98419300
H	2.34977800	1.43889200	-1.04058800
C	2.57104500	0.60231100	0.98062800
H	3.63751400	0.56416700	1.20740400
H	2.15830800	1.52929400	1.39150100
C	1.82973300	-0.59232500	1.57401000
C	0.45854100	-0.48258300	0.86717900
H	-0.19159900	0.21106000	1.40265900
N	-0.20369300	-1.79537200	0.78317900
N	-0.10511100	-2.29506100	-0.33555900
H	1.74327800	-0.55999900	2.66596100
O	2.47812500	-1.76522700	1.13388900
Si	2.75664000	-3.14492900	2.04874200
C	1.21635800	-3.68011700	2.96174500
H	1.45151300	-4.51663900	3.62669100
H	0.80481600	-2.87360400	3.57453100
H	0.43367900	-3.99083800	2.26751400
C	4.11862100	-2.76077600	3.27892100
H	3.81547400	-1.97572600	3.97772700
H	4.37462100	-3.64412000	3.87046900
H	5.02328600	-2.42403700	2.76672600
C	3.29447900	-4.42054700	0.80194900
H	3.51394600	-5.37793400	1.28131700
H	2.49917500	-4.58301700	0.06997200
H	4.18996600	-4.09502700	0.26751100

anti-2b

C	0.44910100	0.10719600	-1.91005400
H	-0.51860800	0.51184800	-2.21891700
H	1.20577400	0.47860200	-2.60372000
C	0.76519200	0.33087300	-0.43651200
H	0.19227100	1.15163000	-0.00517700
C	2.26092200	0.46890500	-0.09750400
H	2.85342600	-0.08974400	-0.83148100
H	2.60784600	1.50292900	-0.11112900
C	2.37696600	-0.19480600	1.27579200
H	3.40486900	-0.43442400	1.55876800
H	1.95005100	0.45226400	2.04950400
C	1.48889200	-1.44288100	1.15449800
C	0.37735900	-1.02438200	0.17527400
H	-0.58900200	-1.06095700	0.68208700
N	0.30330600	-1.95429000	-0.98584100
N	0.35589300	-1.37296800	-2.06611100
H	2.05782600	-2.26131100	0.69164900
O	0.93682800	-1.87461500	2.37529000
Si	1.80897100	-2.57357600	3.62115800
C	0.55519900	-3.49160400	4.64959400
H	-0.21870900	-2.80968500	5.00960800
H	0.06812200	-4.27336800	4.06281500
H	1.02394100	-3.95890900	5.51942100
C	3.09903700	-3.72710000	2.90107400
H	3.86456400	-3.18362400	2.34016000
H	3.60757800	-4.27237300	3.70097000
H	2.64574400	-4.46150000	2.23044400
C	2.64982000	-1.24758700	4.64609100
H	3.14286900	-1.69117000	5.51587900
H	3.40873400	-0.70994000	4.07283100
H	1.92197500	-0.51827400	5.01095600

IN-2c

C	0.74070849	4.03478348	0.36627966
H	1.23543550	4.79477368	-0.23117424
C	1.02050200	2.75863300	0.10897200
H	1.73119861	2.53297529	-0.68327963
C	0.46157500	1.57252500	0.84040500
H	-0.12559300	1.89804600	1.70454700
H	-0.22059200	1.02500000	0.17960600
C	1.57208400	0.62577300	1.30237800
H	2.17056100	0.29377700	0.44617500
H	2.25445600	1.14768400	1.97835000
C	1.04921300	-0.60989400	2.03087500
H	0.41746500	-0.29026100	2.87098700
C	0.16212300	-1.48069100	1.16853400
H	-0.87045100	-1.71736700	1.36990900
N	0.67329400	-2.00114500	0.09669900
N	1.17100300	-2.45019300	-0.81281800
O	2.18124700	-1.30566100	2.52175600
Si	2.12937300	-2.76022500	3.35570500
C	3.75356600	-2.84151400	4.26879800
H	3.84545900	-3.77727500	4.82634600
H	4.58959400	-2.78347400	3.56780600
H	3.84887000	-2.01283400	4.97373400
C	0.67569200	-2.73323500	4.54169900
H	0.71923000	-1.86394000	5.20330900
H	-0.27839800	-2.70820200	4.00795900
H	0.67586400	-3.62855200	5.16953900
C	1.97864800	-4.20881400	2.17800100
H	0.98073700	-4.27979700	1.73877200
H	2.70059200	-4.12127900	1.36177600
H	2.18085100	-5.14761800	2.70169800
C	-0.21002050	4.52113546	1.43219302
H	-1.19776630	4.06547885	1.33034644
H	0.15897311	4.28351694	2.43410323
H	-0.33747313	5.60191547	1.37731676

syn-TS-2c

C	0.79142100	0.12936400	-1.78555600
H	0.00632200	0.47415200	-2.45005300
C	0.98960200	0.82917400	-0.61025500
H	0.36079200	1.70164100	-0.44786100
C	2.30186700	0.83448000	0.15538300

H	2.99141500	0.11076300	-0.27964400
H	2.76989300	1.81959800	0.06978600
C	2.09046200	0.48178000	1.63063000
H	3.03062700	0.29454200	2.15538800
H	1.56653300	1.29251600	2.14950900
C	1.21502600	-0.77137200	1.66341600
H	0.98892200	-1.05971500	2.70018900
C	-0.04511500	-0.37821300	0.90842200
H	-0.79284500	0.21238300	1.42830300
N	-0.54133200	-1.26873700	0.04442500
N	-0.52603600	-1.55959000	-1.06838700
O	1.87868900	-1.80388800	0.98007100
Si	1.82576600	-3.43460600	1.37887000
C	2.54314300	-4.29937900	-0.10553500
H	2.59280100	-5.38022800	0.04894600
H	1.92707600	-4.10911100	-0.98807900
H	3.55344800	-3.93933700	-0.31401800
C	2.86589900	-3.70452500	2.91292500
H	3.88938700	-3.35666600	2.75335300
H	2.45853300	-3.16834300	3.77455300
H	2.90716900	-4.76495800	3.17665200
C	0.06032000	-3.96146800	1.70922900
H	-0.42011800	-3.31409900	2.44874600
H	-0.54035100	-3.93105900	0.79753100
H	0.03665000	-4.98247100	2.10056600
C	1.80824900	-0.80855700	-2.38760700
H	1.33468900	-1.46295000	-3.12153400
H	2.61064600	-0.26260000	-2.89608700
H	2.26439000	-1.44189100	-1.62251400

anti-TS-2c

C	1.49422800	0.23675900	-1.77532500
H	0.87498600	-0.03021200	-2.62502000
C	0.85408800	0.60452000	-0.60757900
H	-0.23164300	0.65402100	-0.63724800
C	1.49659900	1.45016500	0.48031400
H	2.57280900	1.53069000	0.30610900
H	1.09810100	2.46808500	0.43609900
C	1.25325100	0.84076000	1.85933500
H	1.81722200	1.33529600	2.65305500
H	0.19175000	0.89086000	2.12609200
C	1.65038100	-0.63206400	1.77680600
C	0.85078500	-1.19690300	0.62374200
H	-0.16498600	-1.51694500	0.84187400
N	1.48097200	-1.96606000	-0.27724700
N	1.93613100	-1.94551600	-1.33271700
H	2.71658900	-0.69627500	1.51339600
O	1.41269500	-1.26358100	3.01253800
Si	1.83912300	-2.85042900	3.36234600
C	0.55139200	-4.03789500	2.69577200
H	-0.45057400	-3.74765500	3.02248600
H	0.55940500	-4.07427200	1.60384000
H	0.74126500	-5.05023600	3.06437100
C	3.50720700	-3.21769200	2.59211600
H	4.26610200	-2.50465500	2.92535100
H	3.84385500	-4.21808800	2.87773800
H	3.46421500	-3.18784100	1.49953500
C	1.90023600	-2.93473300	5.22364000
H	2.16158000	-3.94033700	5.56312700
H	2.64010400	-2.23705100	5.62198900
H	0.92932600	-2.67568800	5.65240800
C	2.93863500	0.54558100	-2.08595300
H	3.28146500	-0.05576700	-2.92898300
H	3.08153500	1.60010600	-2.34548200
H	3.59781000	0.32306200	-1.24241700

syn-2c

C	0.56990500	-1.38744000	-1.36600400
H	-0.19028500	-1.21949500	-2.13374000
C	0.85413300	-0.09132400	-0.57363100
H	0.17486100	0.69141700	-0.91195100
C	2.30093200	0.45286300	-0.49581600
H	3.01099800	-0.25491900	-0.92226400
H	2.41070200	1.39708600	-1.02986200
C	2.58020000	0.59703300	1.01105600
H	3.64186400	0.58207800	1.26249500
H	2.14041700	1.52069200	1.40093100
C	1.84855400	-0.60384700	1.60343600
C	0.49520500	-0.53192800	0.86133400
H	-0.18971800	0.13509200	1.38836200
N	-0.13016900	-1.85998300	0.75541300
N	-0.07328800	-2.31555400	-0.38354400
H	1.73317800	-0.55546600	2.69212500
O	2.53240200	-1.77108200	1.20264100
Si	2.80096400	-3.13264400	2.14669500
C	1.24796300	-3.66670900	3.03799600
H	1.47700200	-4.48923900	3.72223600
H	0.81675000	-2.85355500	3.62811200

H	0.48277500	-3.99602500	2.33306400
C	4.13493400	-2.71717100	3.39693400
H	3.81092200	-1.92210200	4.07468200
H	4.38565800	-3.58742800	4.00978500
H	5.04728500	-2.38184800	2.89763500
C	3.37410300	-4.42430800	0.93137000
H	3.61518900	-5.36389800	1.43495300
H	2.58665600	-4.62397300	0.19983700
H	4.26434700	-4.09033100	0.39276300
C	1.74628500	-2.12183300	-1.99718100
H	1.39221500	-3.04931600	-2.44980800
H	2.20916800	-1.51102500	-2.77488500
H	2.49391300	-2.36523600	-1.24061900

anti-2c

C	0.50083800	0.14117300	-1.89597300
C	0.86515500	0.30202400	-0.42059000
H	0.30241400	1.10676500	0.05441400
C	2.36303500	0.42114000	-0.08289200
H	2.95590000	-0.15545200	-0.80088200
H	2.72928500	1.44853300	-0.10051500
C	2.45625400	-0.23966100	1.29422200
H	3.47941600	-0.48288900	1.59098600
H	2.02208200	0.41100200	2.06077300
C	1.56304000	-1.48481100	1.16380200
C	0.49093500	-1.07487200	0.14065100
H	-0.50146700	-1.15624500	0.58982800
N	0.50881700	-1.96866200	-1.05559000
N	0.52800200	-1.34059300	-2.11035300
H	2.13907300	-2.31751000	0.73683600
O	0.96556300	-1.88706200	2.37459200
Si	1.78774000	-2.58361000	3.65504100
C	0.48501200	-3.44661100	4.67049300
H	-0.27910600	-2.73734000	4.99722700
H	-0.00824800	-4.22827800	4.08882600
H	0.92054300	-3.90656100	5.56122700
C	3.06338200	-3.78432800	2.98737000
H	3.85465600	-3.26886800	2.43571700
H	3.53969600	-4.32875100	3.80737800
H	2.60710600	-4.51779000	2.31773900
C	2.64114500	-1.26212600	4.67568700
H	3.10240600	-1.70287900	5.56413500
H	3.42731300	-0.75570100	4.11059700
H	1.92567900	-0.50686400	5.01093000
H	-0.54765100	0.42184700	-2.05364600
C	1.36572400	0.84210600	-2.92917900
H	0.99878700	0.62524600	-3.93306200
H	1.34318900	1.92305600	-2.77620800
H	2.40211000	0.50615600	-2.86940700

IN-3a

C	1.25616600	3.84611100	-0.02967900
H	0.76023800	4.35485800	0.79150000
H	1.78675400	4.46282300	-0.74508000
C	1.20958700	2.52440700	-0.14875000
H	1.72054300	2.04656200	-0.98324700
C	0.50996900	1.60208200	0.80679400
H	-0.01685100	2.19562300	1.55895300
H	-0.24624200	1.02228800	0.26371400
C	1.49811000	0.64386600	1.47760200
H	2.12488900	0.16078200	0.71925300
H	2.18072900	1.20263700	2.12326000
C	0.86521800	-0.45821300	2.33811900
C	-0.01541700	-1.37458300	1.51121300
H	-0.95436900	-1.78766300	1.84811300
N	0.36260100	-1.71542000	0.32035500
N	0.73430700	-1.98524600	-0.71254700
O	1.97308300	-1.14389200	2.92008600
Si	2.11555300	-2.74270300	3.38855900
C	3.71632200	-2.78929900	4.34696800
H	3.92232900	-3.79655800	4.71871800
H	4.55388000	-2.48383400	3.71562100
H	3.67679800	-2.11153500	5.20261300
C	0.68377900	-3.26648400	4.48548800
H	0.61516900	-2.63938300	5.37818100
H	-0.27358500	-3.22199600	3.95970500
H	0.82798800	-4.29950000	4.81564300
C	2.22088400	-3.88810200	1.90729400
H	1.24753300	-4.06016400	1.44190100
H	2.89221900	-3.48178800	1.14597400
H	2.61802800	-4.86040400	2.21366100
C	0.00931100	0.12135800	3.46386800
H	-0.84651200	0.67208100	3.06974700
H	-0.36095600	-0.68225600	4.10407900
H	0.62249500	0.79084000	4.06984700

syn-TS-3a

C	0.63913700	0.06806000	-1.77771800
H	-0.17476800	0.36956800	-2.42440300
H	1.35534400	-0.63043800	-2.19668800
C	0.94719500	0.77534900	-0.63762600
H	0.38050400	1.68210800	-0.43510200
C	2.31468000	0.70353300	0.01443400
H	2.87165500	-0.13214600	-0.41490200
H	2.87610400	1.61873900	-0.19561300
C	2.18726000	0.49685900	1.52167300
H	3.15005300	0.30833700	2.00485600
H	1.74016000	1.38221800	1.98903600
C	1.25826400	-0.70476000	1.75288300
C	-0.00648900	-0.34140500	0.97012000
H	-0.74617600	0.28076800	1.46601800
N	-0.53052000	-1.28161500	0.17810800
N	-0.57103400	-1.64592700	-0.91058100
O	1.87367000	-1.81717500	1.14760800
Si	1.81311000	-3.46888700	1.42611300
C	2.73788600	-4.18274400	-0.02604200
H	2.78149300	-5.27361000	0.02882600
H	2.23970000	-3.90939200	-0.95961000
H	3.76098400	-3.80206800	-0.06446000
C	2.68023500	-3.88516500	3.03689400
H	3.63633100	-3.36074100	3.11298200
H	2.07806200	-3.62607400	3.91073400
H	2.88437400	-4.95882900	3.08349000
C	0.06123800	-4.13417100	1.47315300
H	-0.56715800	-3.62102600	2.20525800
H	-0.42424400	-4.04820200	0.49870200
H	0.08751900	-5.19370100	1.74684700
C	0.98765500	-0.92663000	3.23908500
H	0.30877700	-1.76877700	3.39402300
H	1.92592800	-1.12798500	3.76160400
H	0.53045300	-0.03663900	3.67676000

anti-TS-3a

C	1.37209000	0.24037500	-1.82254400
H	0.85350700	-0.14953700	-2.68842100
H	2.32990300	0.71399000	-2.01221800
C	0.71951800	0.43880800	-0.62559100
H	-0.34892300	0.23639600	-0.59861900
C	1.21038800	1.41694900	0.42937200
H	2.20839500	1.77207300	0.15790000
H	0.56257400	2.29806000	0.44241000
C	1.23151700	0.76061400	1.80831600
H	1.72867000	1.36871600	2.56837000
H	0.20953500	0.57370800	2.15504300
C	1.93493100	-0.60251600	1.69985800
C	1.19513100	-1.29474700	0.56316000
H	0.26604600	-1.79667500	0.81571800
N	1.91229700	-1.92487900	-0.38111800
N	2.33209700	-1.80204500	-1.44279800
O	1.77445600	-1.25464500	2.94734600
Si	1.87952200	-2.87096800	3.36769600
C	0.36682000	-3.81656400	2.78235900
H	-0.55188600	-3.26234100	2.99215100
H	0.40380000	-4.03476700	1.71244200
H	0.30252500	-4.77353000	3.30887100
C	3.40826800	-3.69196400	2.65868700
H	4.32620900	-3.19231700	2.97762700
H	3.45578400	-4.72859500	3.00616000
H	3.38809600	-3.71396000	1.56550500
C	1.93729300	-2.84543200	5.23180100
H	1.99361500	-3.85820300	5.63909900
H	2.80800700	-2.28768000	5.58386100
H	1.04436300	-2.36512700	5.63837500
C	3.42069200	-0.43202700	1.39143100
H	3.91952300	-1.40042700	1.33912800
H	3.57836500	0.07884500	0.43983300
H	3.87680500	0.15486100	2.19035500

syn-3a

C	0.47721800	-1.54827500	-1.12343900
H	-0.18345500	-1.56381300	-1.99130500
H	1.38448700	-2.10576400	-1.37338000
C	0.76720500	-0.15616100	-0.55507300
H	0.08503200	0.57925200	-0.98278700
C	2.23353000	0.33831400	-0.61187300
H	2.88305300	-0.45014300	-0.99602100
H	2.34999300	1.21225700	-1.25340600
C	2.59901100	0.63094700	0.85133600
H	3.67075400	0.58619700	1.05452800
H	2.22562300	1.61505900	1.15410000
C	1.84814800	-0.45469900	1.62808000
C	0.45834900	-0.39298200	0.93626900
H	-0.16318200	0.36995500	1.40875100
N	-0.22770300	-1.69075900	1.02632000
N	-0.19512700	-2.31024100	-0.03510400
O	2.45713400	-1.68149200	1.26785700
Si	2.74042200	-3.13188100	2.05760100
C	1.19884800	-3.94561700	2.73429600
H	1.48150000	-4.87899500	3.23200900
H	0.67704900	-3.32212500	3.46331300
H	0.49098000	-4.17520600	1.93577600
C	3.97115500	-2.87459300	3.45357700
H	3.50971400	-2.41395200	4.33020700
H	4.38283300	-3.83801700	3.76858200
H	4.80512400	-2.24417400	3.13400500
C	3.48840900	-4.19239500	0.71510000
H	3.77340900	-5.17568800	1.09819600
H	2.76574000	-4.34373800	-0.09105100
H	4.37805700	-3.72087100	0.29148300
C	1.80281900	-0.23572300	3.13017100
H	1.21795000	-1.01858200	3.61984500
H	2.81399800	-0.23789500	3.54313300
H	1.33939000	0.72674600	3.35695700

anti-3a

C	0.24425900	0.33507400	-1.74362100
H	-0.72722500	0.81558000	-1.88615600
H	0.94221100	0.76952200	-2.46234400
C	0.72684500	0.34258300	-0.29896500
H	0.20849100	1.08628400	0.30642000
C	2.24725100	0.45012100	-0.09761000
H	2.76751100	0.00833300	-0.95467300
H	2.59201200	1.48047400	0.00074600
C	2.48330900	-0.38819800	1.15870600
H	3.53339100	-0.63841100	1.33538500
H	2.10681100	0.15418100	2.03146700
C	1.60998900	-1.64441500	0.95557200
C	0.39430400	-1.09021300	0.15496600
H	-0.50006700	-1.18785900	0.77290700
N	0.12530300	-1.83444600	-1.10391100
N	0.05676200	-1.10292100	-2.08794000
O	1.12213300	-2.17297600	2.17532300
Si	1.87621300	-2.60357900	3.59975400
C	0.52307500	-3.41257000	4.59619800
H	-0.30377700	-2.71733100	4.75833300
H	0.12842500	-4.28578000	4.07193800
H	0.88958800	-3.73822300	5.57310500
C	3.29633700	-3.80060500	3.33114500
H	4.15776200	-3.32265700	2.85752500
H	3.63103800	-4.18646200	4.29890700
H	2.99632900	-4.65254900	2.71632000
C	2.53612800	-1.09897700	4.50800200
H	2.87018900	-1.39254300	5.50773700
H	3.38807700	-0.64487800	3.99663800
H	1.76321000	-0.33529300	4.62740900
C	2.36257300	-2.72676200	0.18402300
H	3.24284300	-3.03654300	0.74907500
H	1.71588700	-3.58795100	0.01751900
H	2.69025400	-2.35940200	-0.79116300

5. Select ^{1}H-NMR and ^{13}C-NMR-Spectra

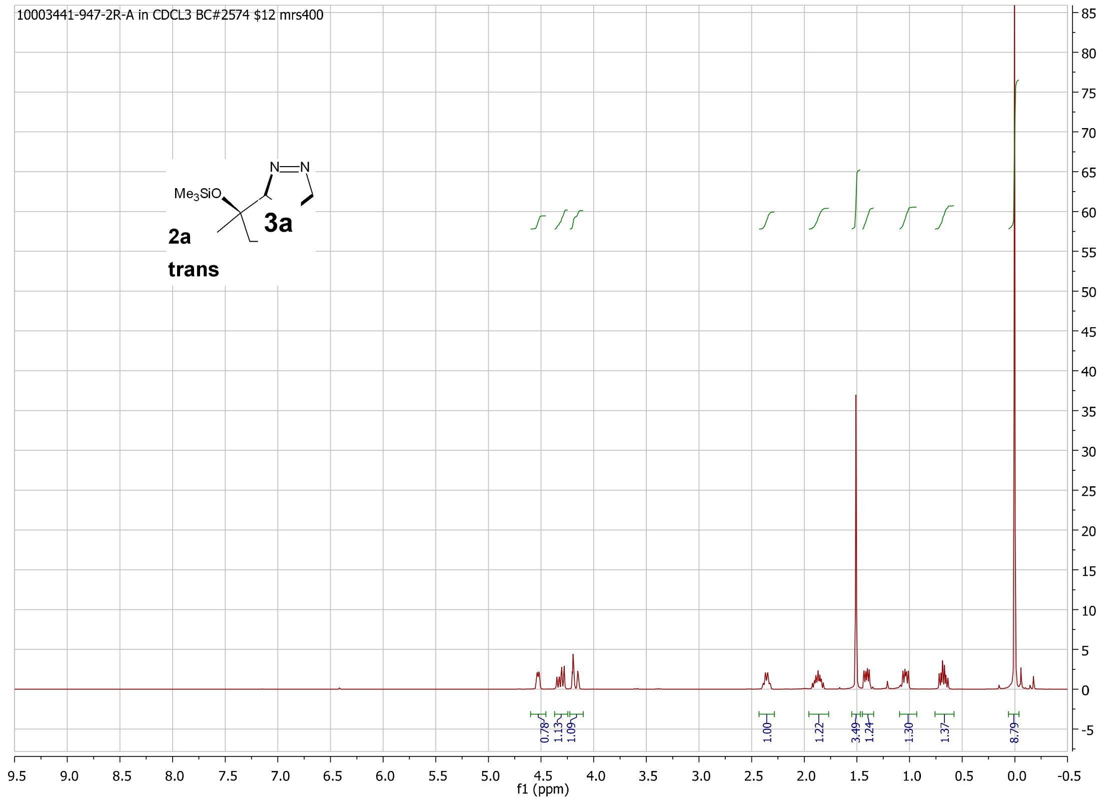

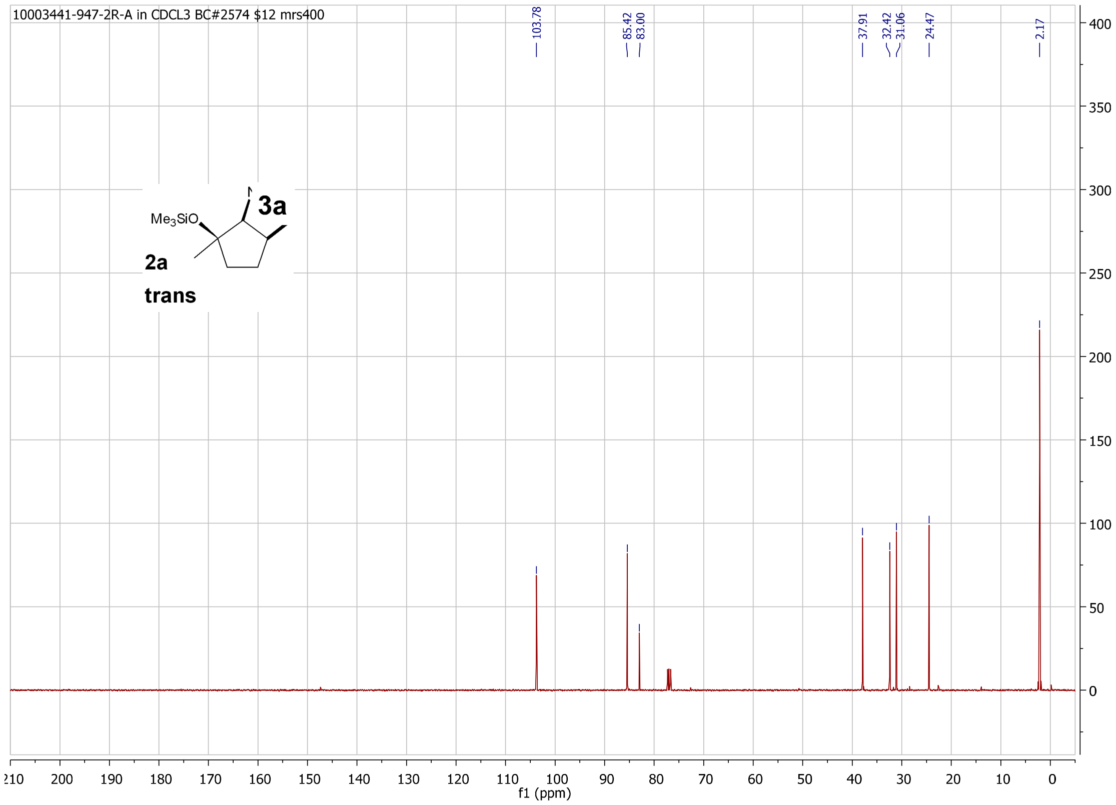

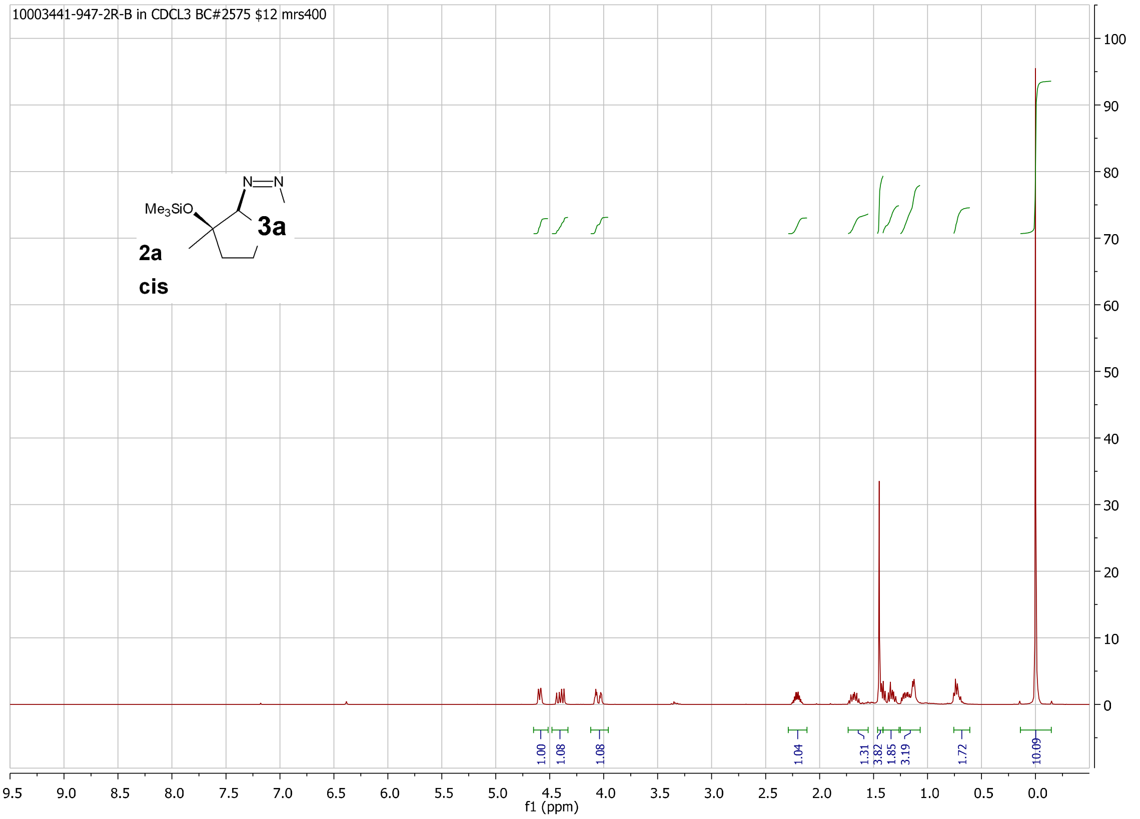
10003441-947-2R-B in CDCL3 BC#2575 $12 mrs400
Me3SiO
N=N
3a
2a
cis
1.00
1.08
1.08
1.04
1.31
3.82
1.85
3.19
1.72
10.09
f1 (ppm)

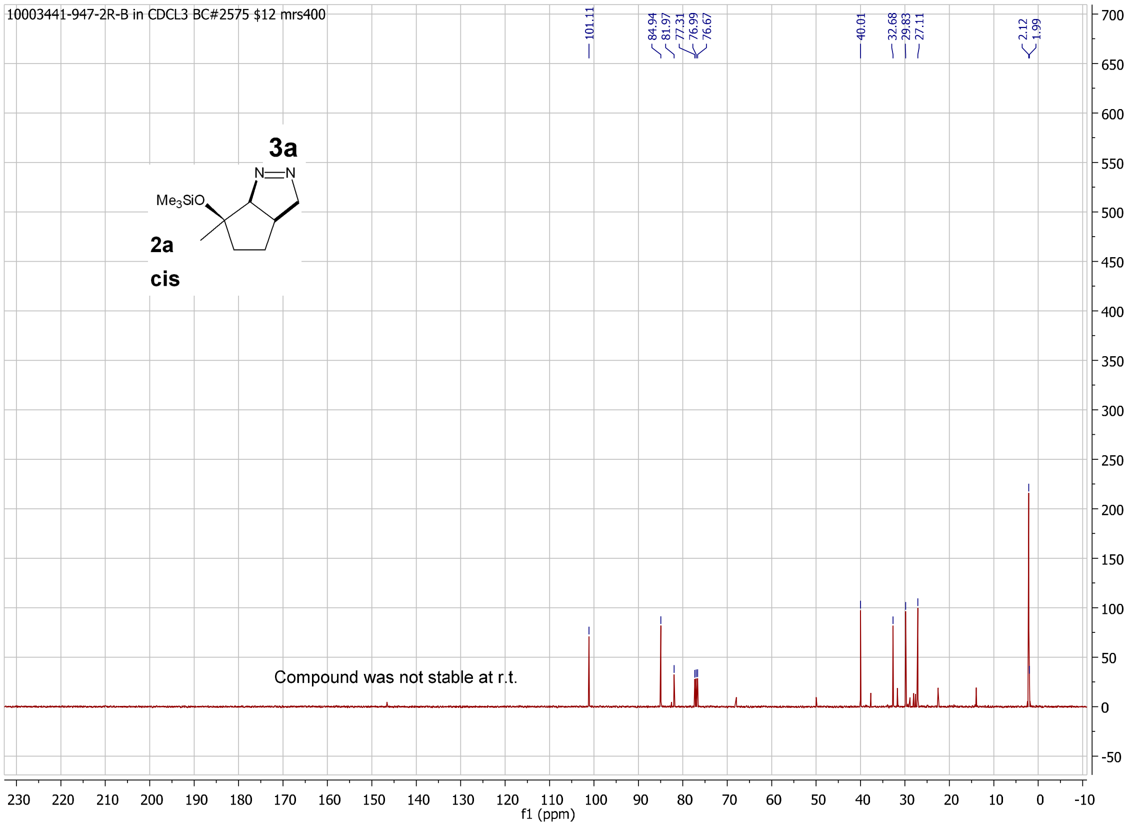
10003441-947-2R-B in CDCL3 BC#2575 $12 mrs400
101.11
84.94
81.97
77.31
76.99
76.67
40.01
32.68
29.83
27.11
2.12
1.99
3a
Me3SiO
N=N
2a
cis
Compound was not stable at r.t.
f1 (ppm)

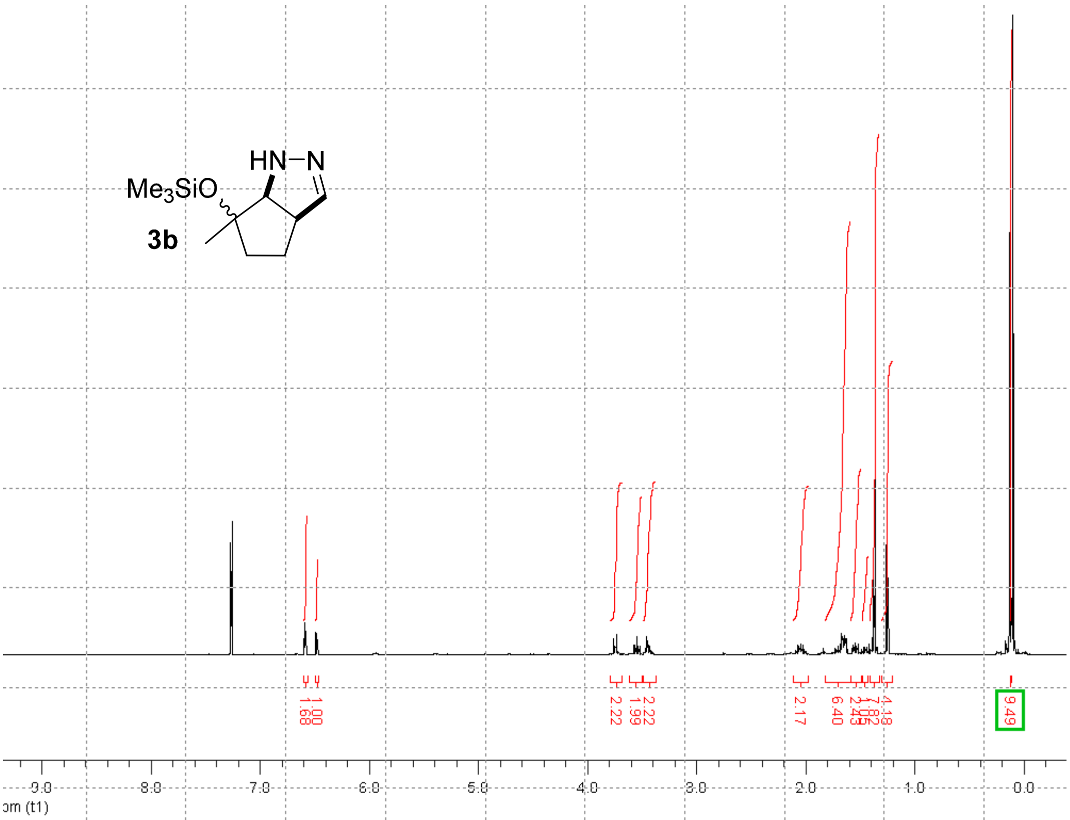
HN–N
Me3SiO
3b
1.68
1.00
2.22
1.99
2.22
2.17
6.40
2.43
1.82
4.18
9.49
9.0
8.0
7.0
6.0
5.0
4.0
3.0
2.0
1.0
0.0
pm (t1)

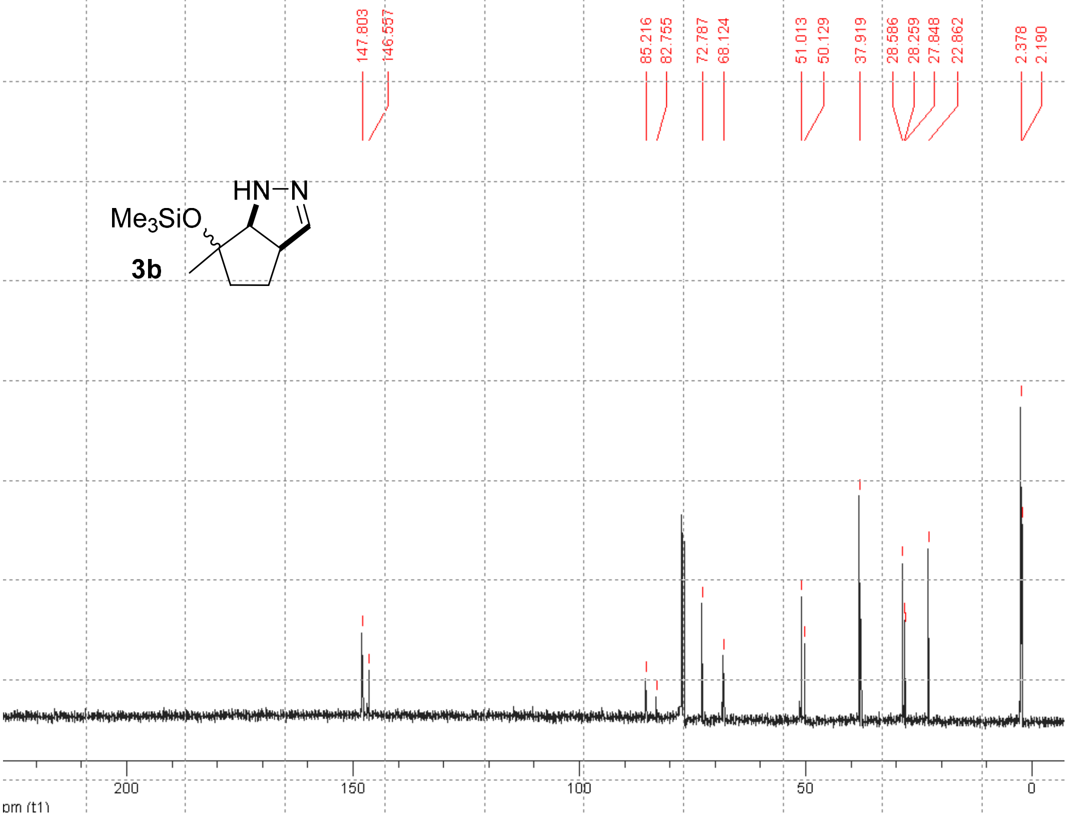
147.803
146.557
85.216
82.755
72.787
68.124
51.013
50.129
37.919
28.586
28.259
27.848
22.862
2.378
2.190
HN–N
Me3SiO
3b
200
150
100
50
0
pm (t1)

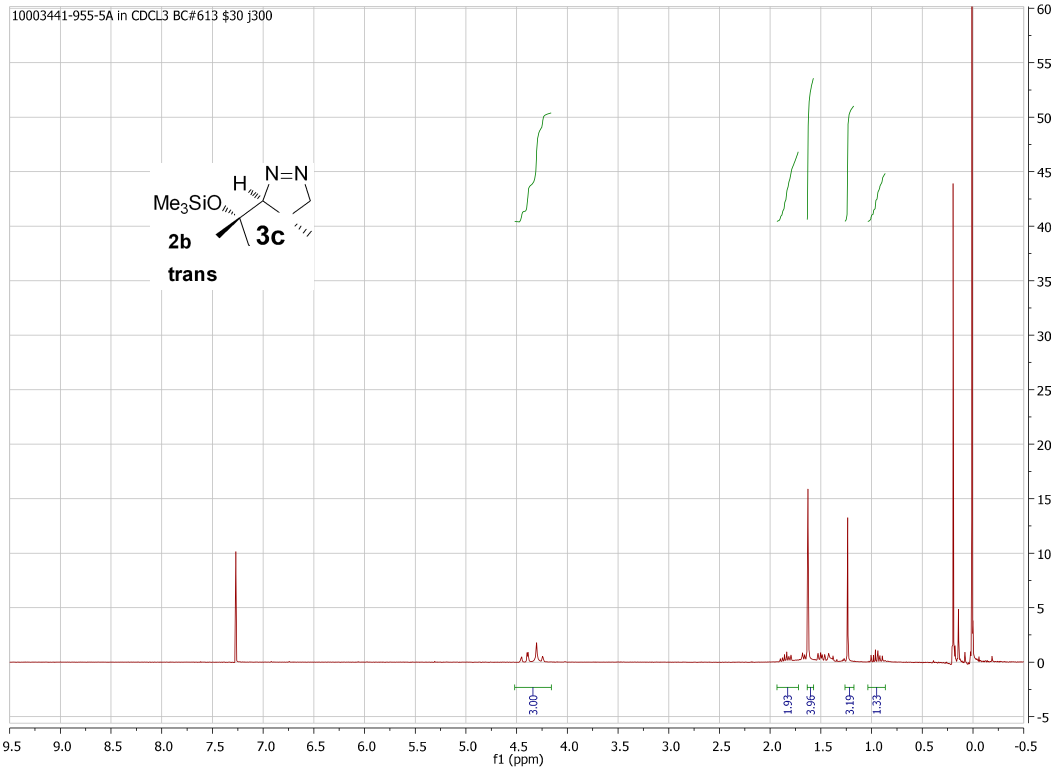
10003441-955-5A in CDCL3 BC#613 $30 j300
H
N=N
Me3SiO
2b
3c
trans
3.00
1.93
3.96
3.19
1.33
f1 (ppm)

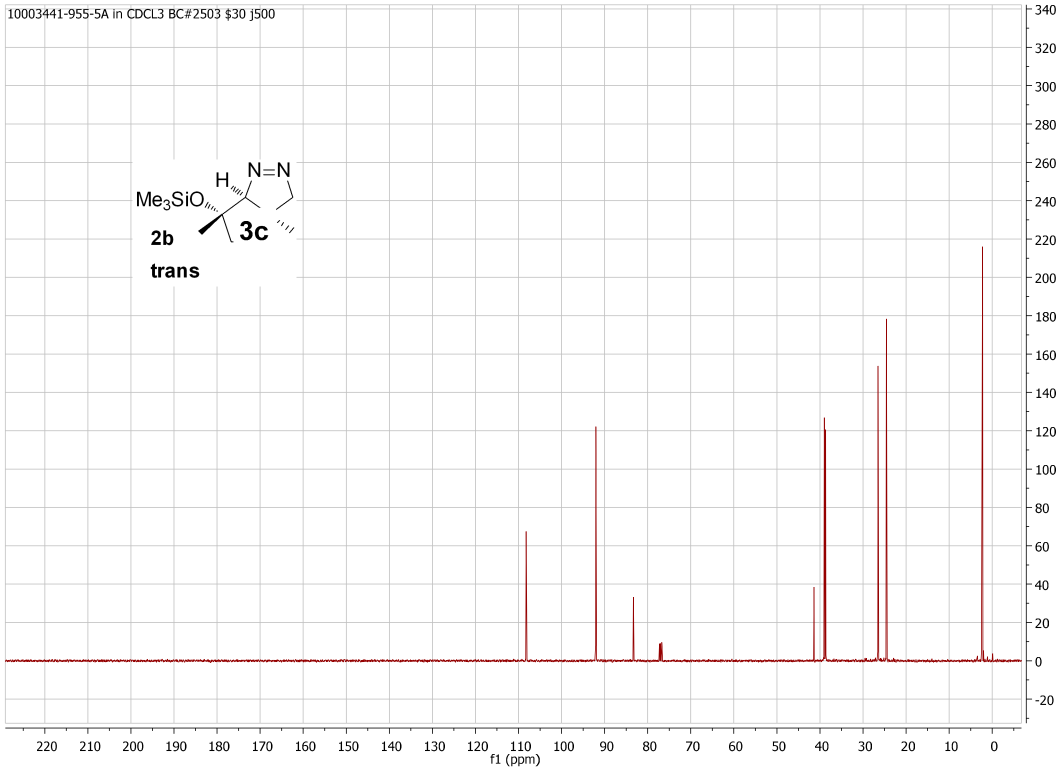
10003441-955-5A in CDCL3 BC#2503 $30 j500
H
N=N
Me3SiO
2b
3c
trans
f1 (ppm)

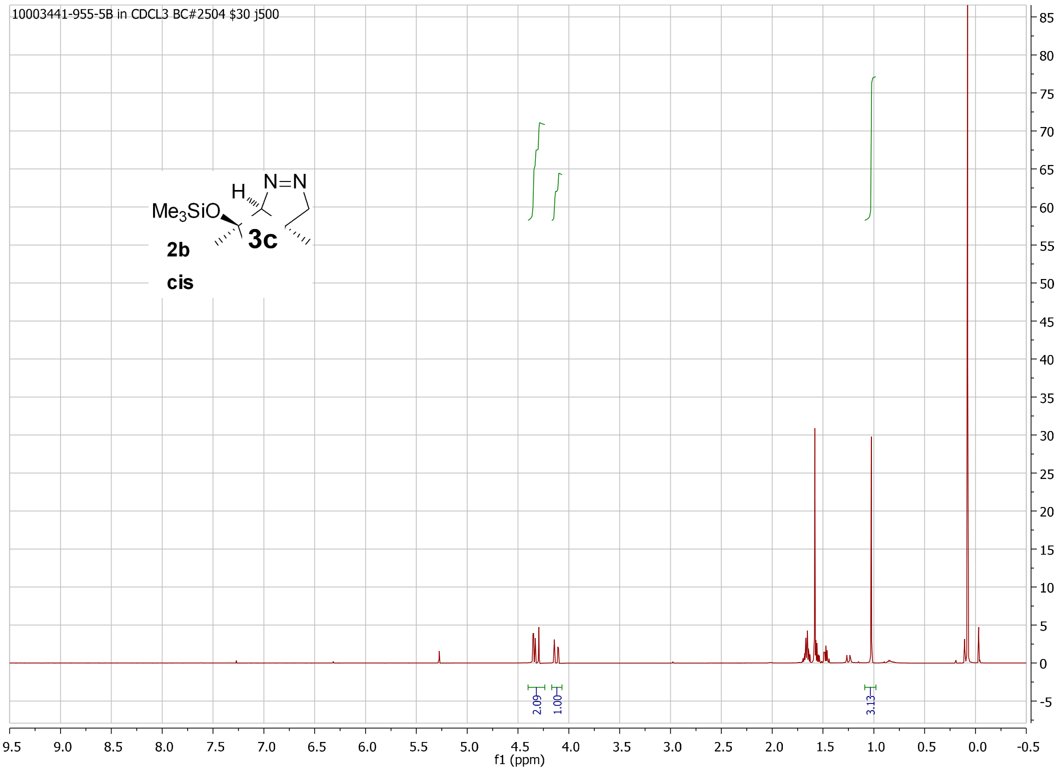
10003441-955-5B in CDCL3 BC#2504 $30 j500
Me3SiO
H
N=N
2b
3c
cis
2.09
1.00
3.13
f1 (ppm)

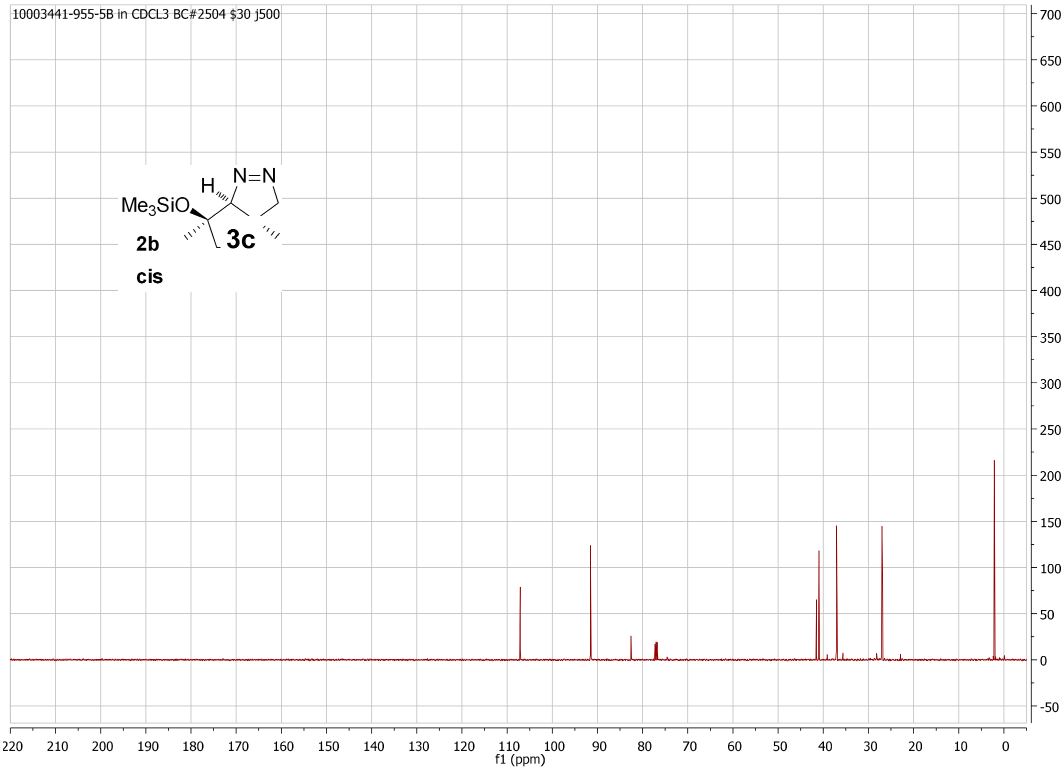
10003441-955-5B in CDCL3 BC#2504 $30 j500
Me3SiO
H
N=N
2b
3c
cis
f1 (ppm)

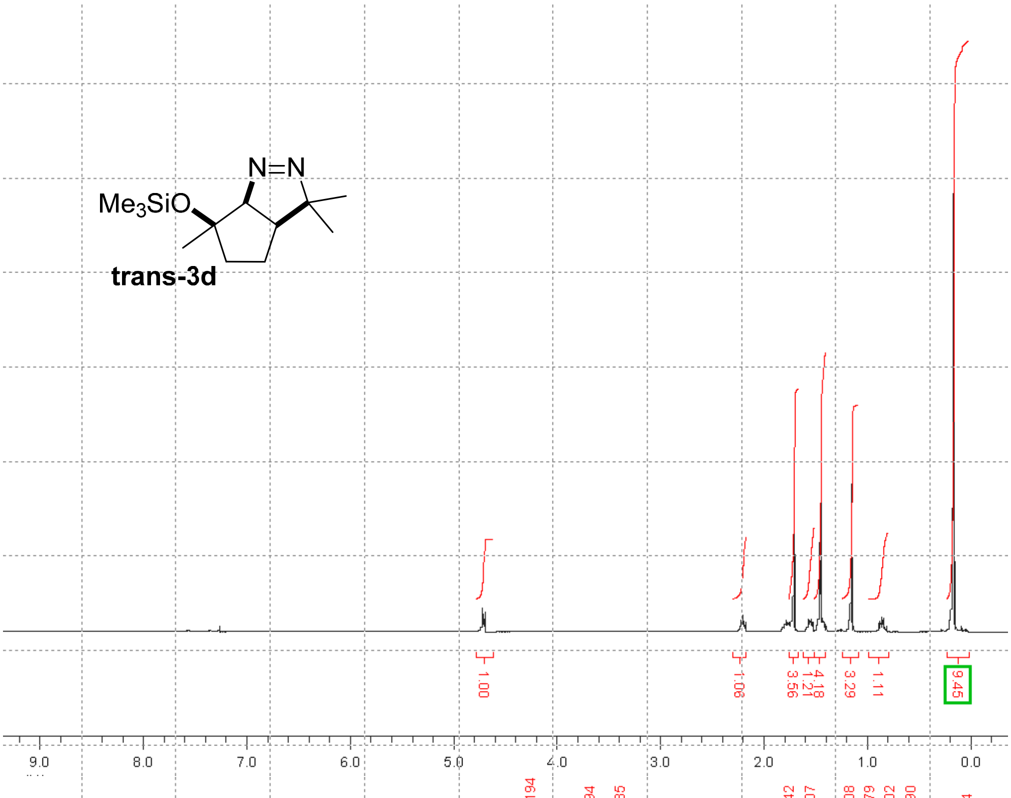
Me3SiO
N=N
trans-3d
1.00
1.08
3.56
1.21
4.18
3.29
1.11
9.45
9.0
8.0
7.0
6.0
5.0
4.0
3.0
2.0
1.0
0.0

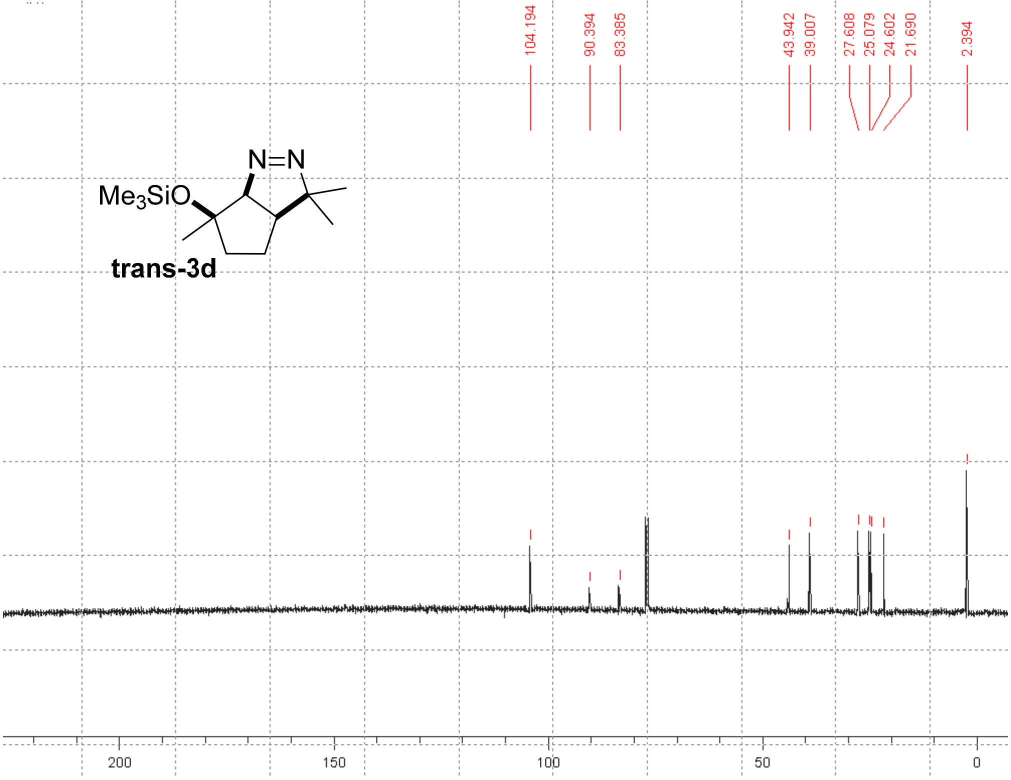
104.194
90.394
83.385
43.942
39.007
27.608
25.079
24.602
21.690
2.394
Me3SiO
N=N
trans-3d
200
150
100
50
0

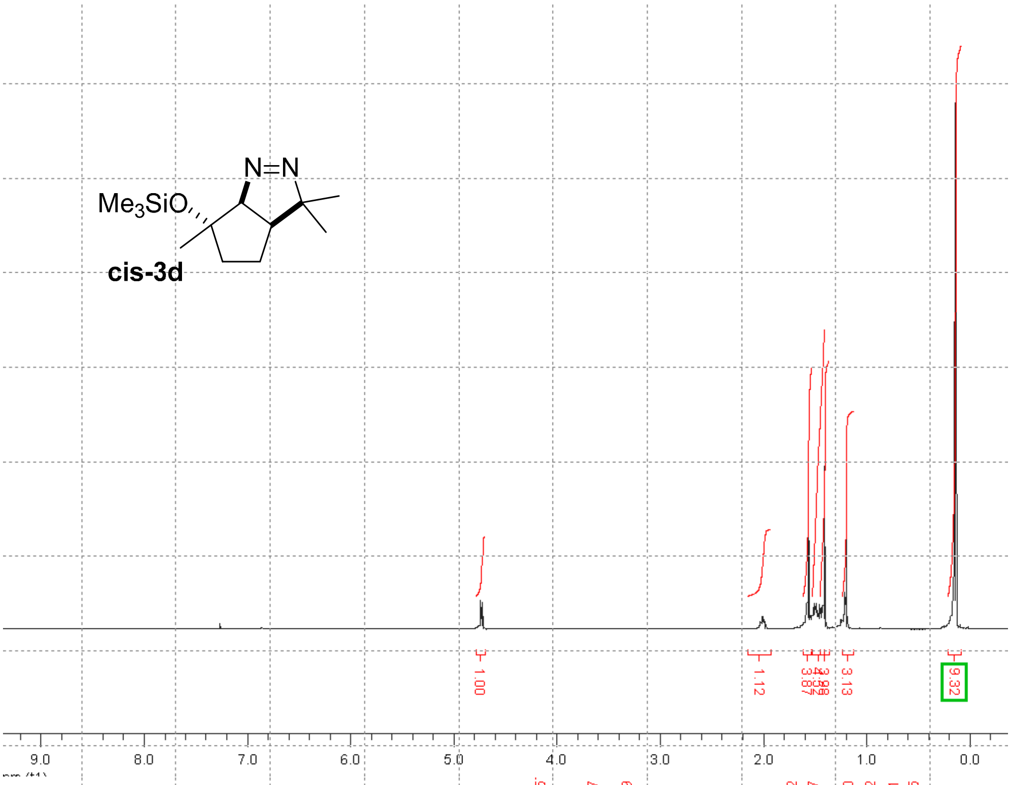
N=N
Me3SiO
cis-3d
1.00
1.12
3.87
3.13
9.32
9.0
8.0
7.0
6.0
5.0
4.0
3.0
2.0
1.0
0.0

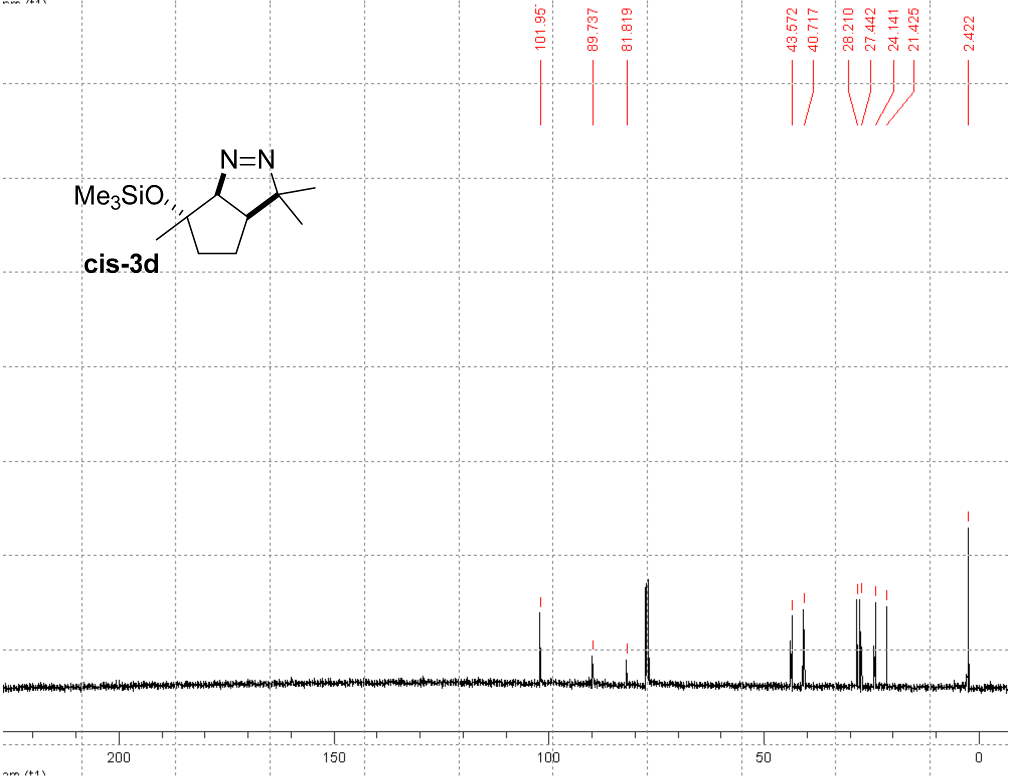
101.95
89.737
81.819
43.572
40.717
28.210
27.442
24.141
21.425
2.422
N=N
Me3SiO
cis-3d
200
150
100
50
0

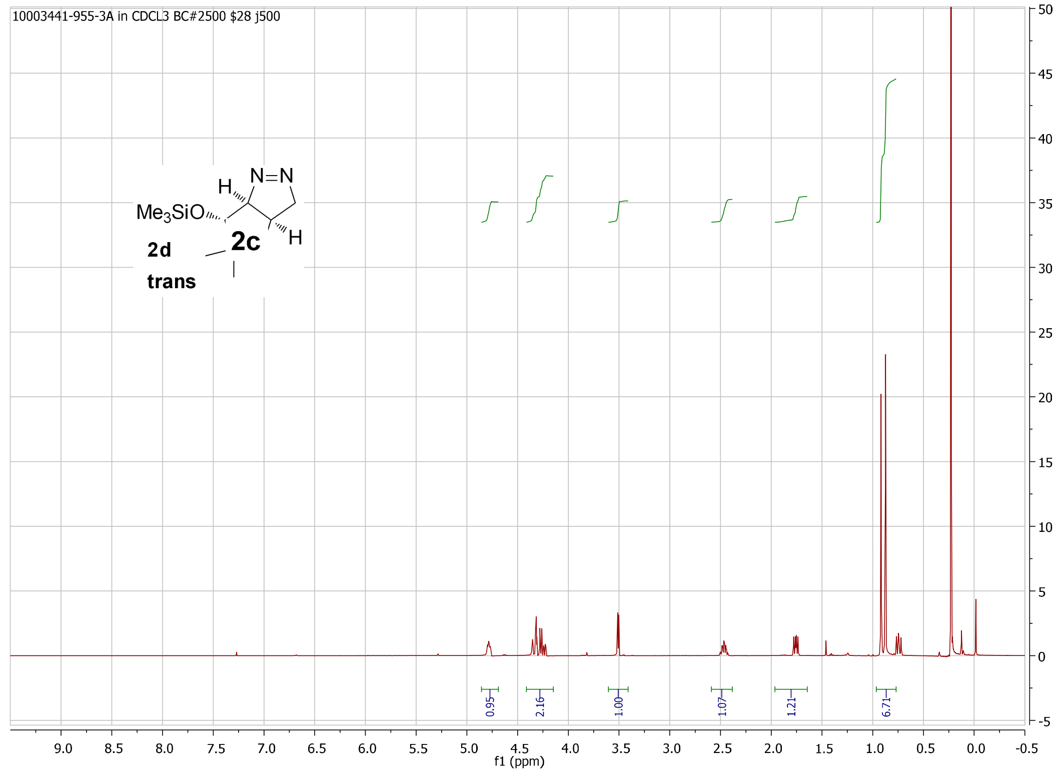

10003441-955-3A in CDCL3 BC#2500 $28 j500
Me3SiO
N=N
H
2d
2c
trans
0.95
2.16
1.00
1.07
1.21
6.71
f1 (ppm)

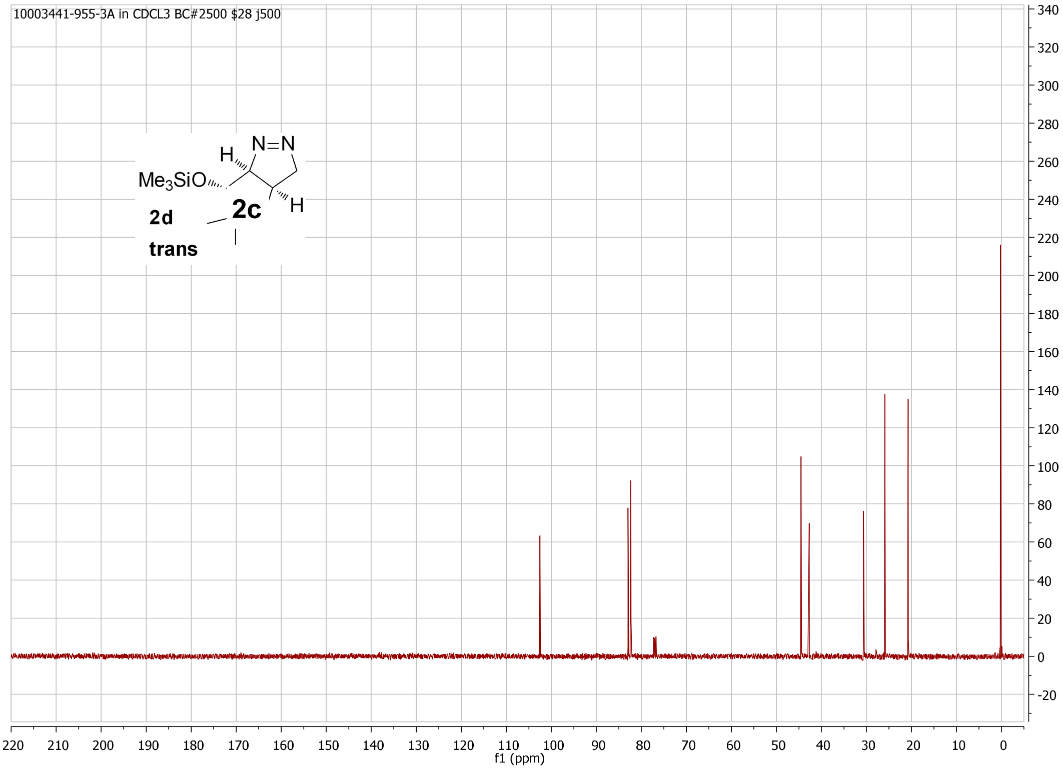

10003441-955-3A in CDCL3 BC#2500 $28 j500
Me3SiO
N=N
H
2d
2c
trans
f1 (ppm)

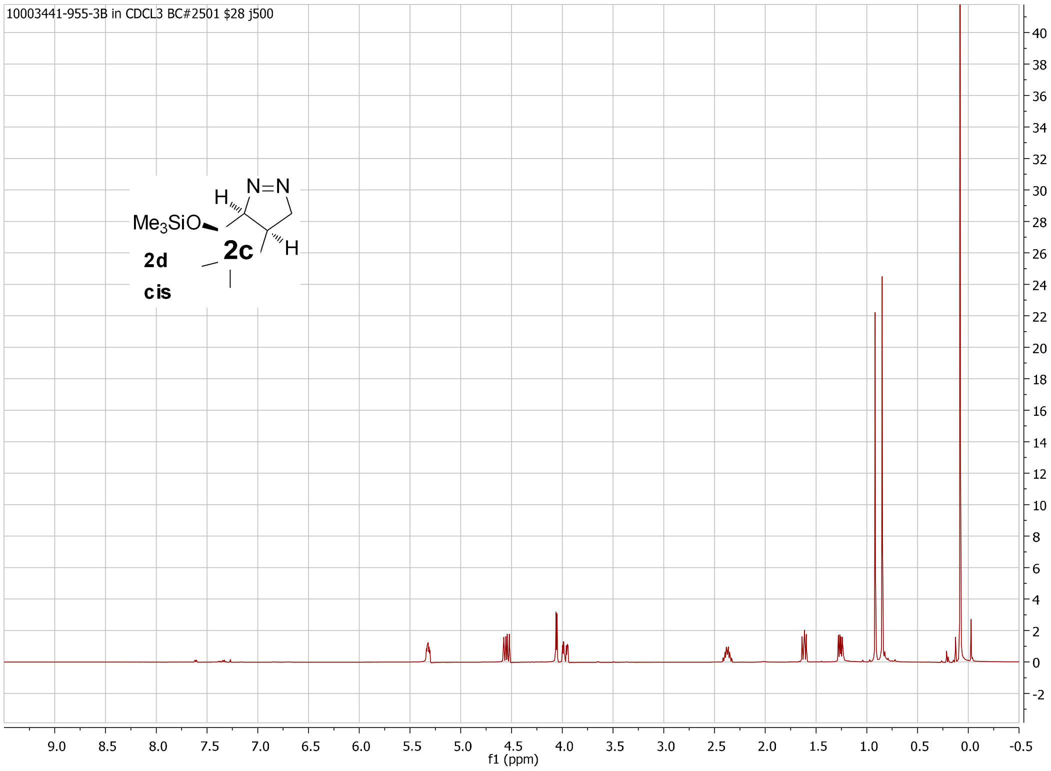

10003441-955-3B in CDCL3 BC#2501 $28 j500
Me3SiO
H
N=N
H
2c
2d
cis
f1 (ppm)

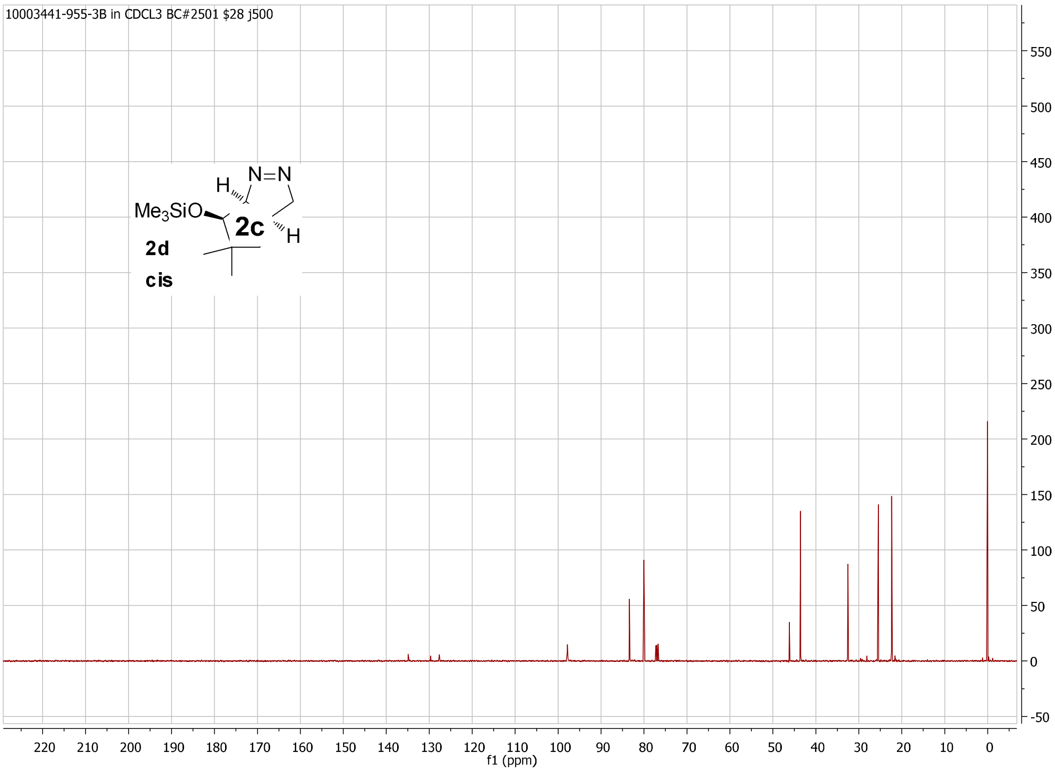

10003441-955-3B in CDCL3 BC#2501 $28 j500
Me3SiO
H
N=N
H
2c
2d
cis
f1 (ppm)

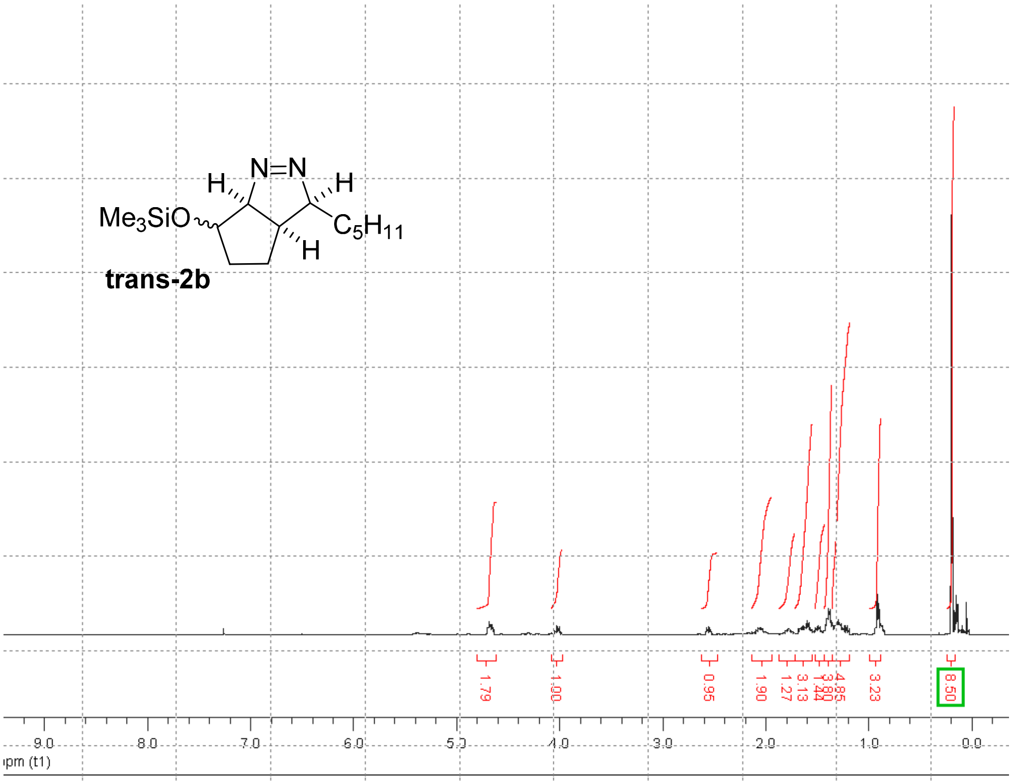
N=N
H
H
Me3SiO
C5H11
H
trans-2b
1.79
1.00
0.95
1.90
1.27
3.13
1.44
3.80
4.85
3.23
8.50
9.0
8.0
7.0
6.0
5.0
4.0
3.0
2.0
1.0
0.0
ppm (t1)

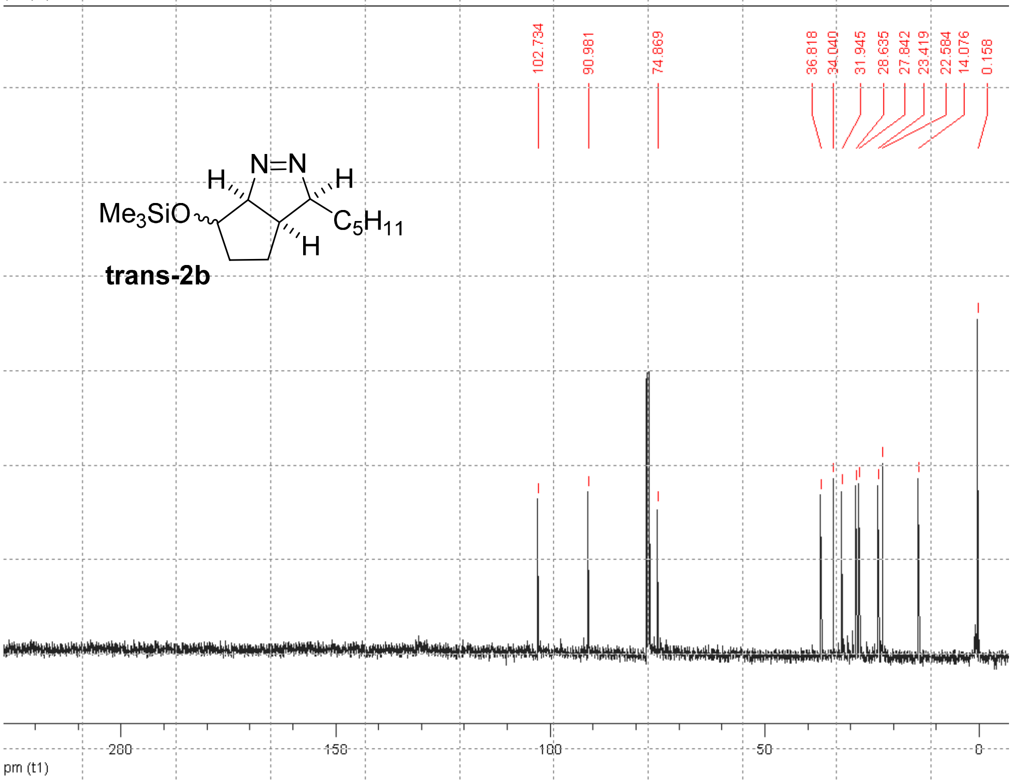
102.734
90.981
74.869
36.818
34.040
31.945
28.635
27.842
23.419
22.584
14.076
0.158
N=N
H
H
Me3SiO
C5H11
H
trans-2b
200
150
100
50
0
ppm (t1)

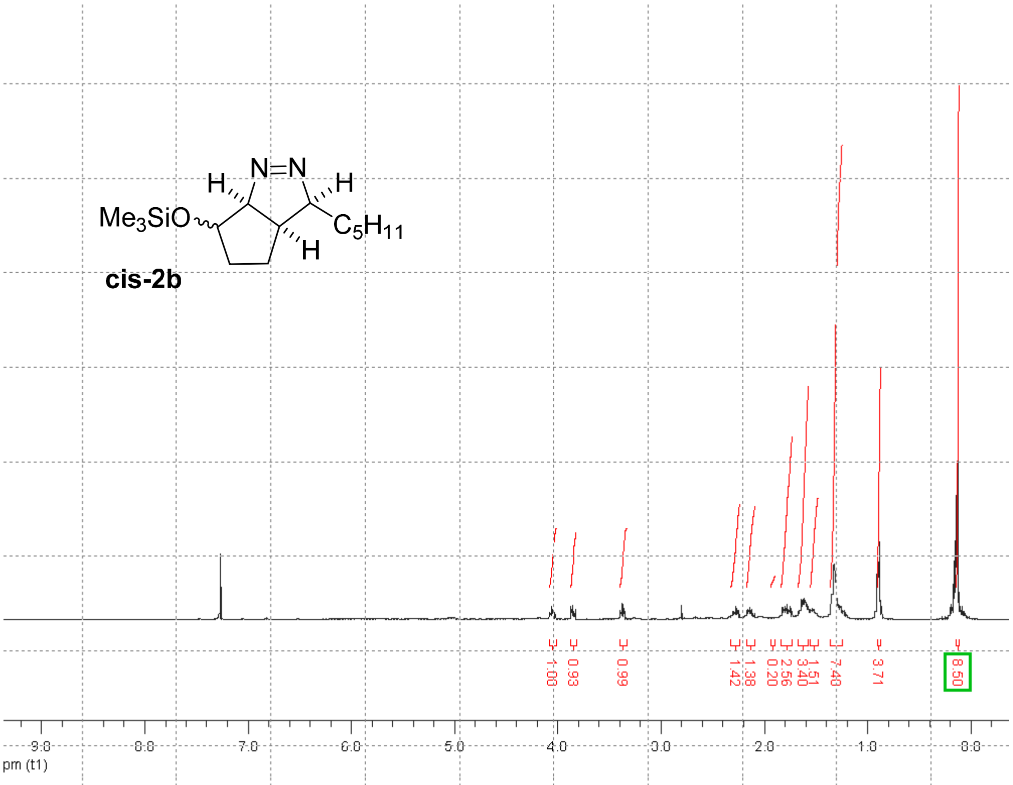

Me3SiO
N=N
H
H
H
C5H11
cis-2b
1.00
0.93
0.99
1.42
1.38
0.20
2.56
3.40
1.51
7.40
3.71
8.50
9.0
8.0
7.0
6.0
5.0
4.0
3.0
2.0
1.0
0.0
pm (t1)

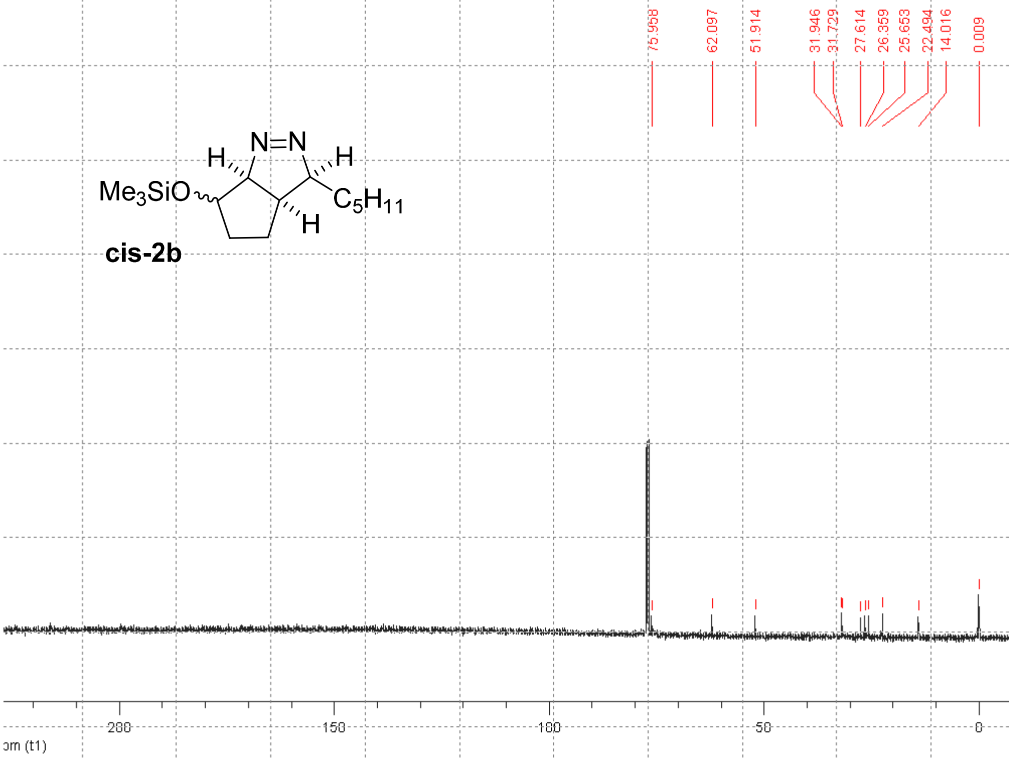

75.958
62.097
51.914
31.946
31.729
27.614
26.359
25.653
22.494
14.016
0.009
Me3SiO
N=N
H
H
H
C5H11
cis-2b
200
150
100
50
0
om (t1)

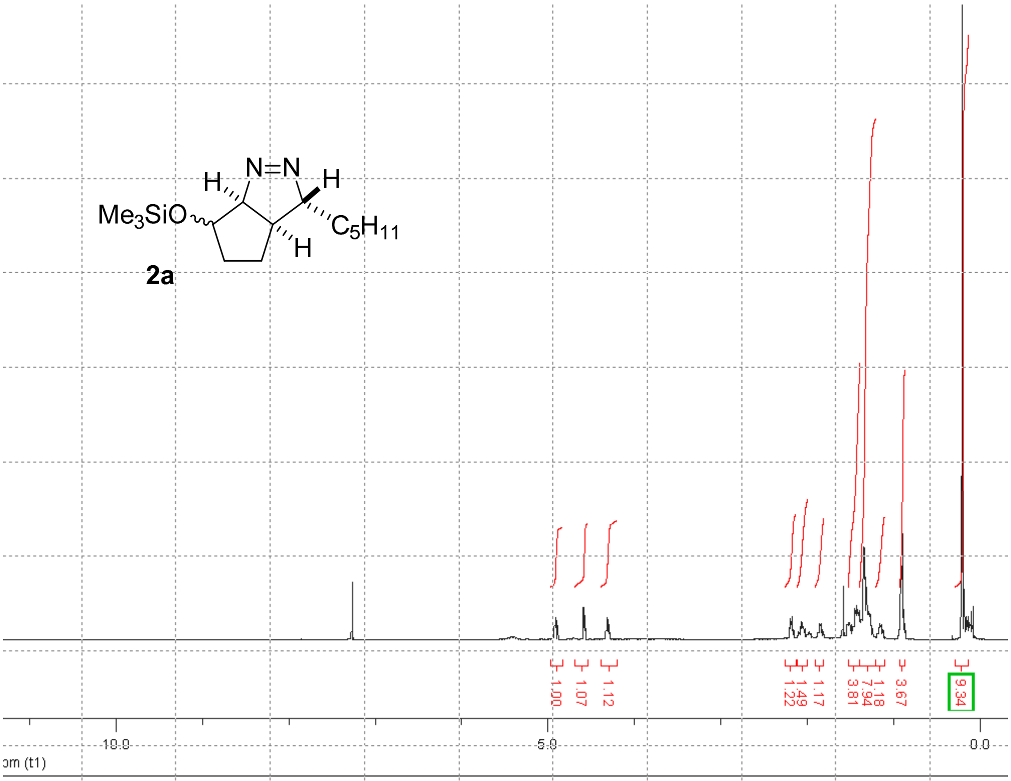

N=N
H
H
Me3SiO
C5H11
H
2a
1.00
1.07
1.12
1.22
1.49
1.17
3.81
7.94
1.18
3.67
9.34
10.0
5.0
0.0
ppm (t1)

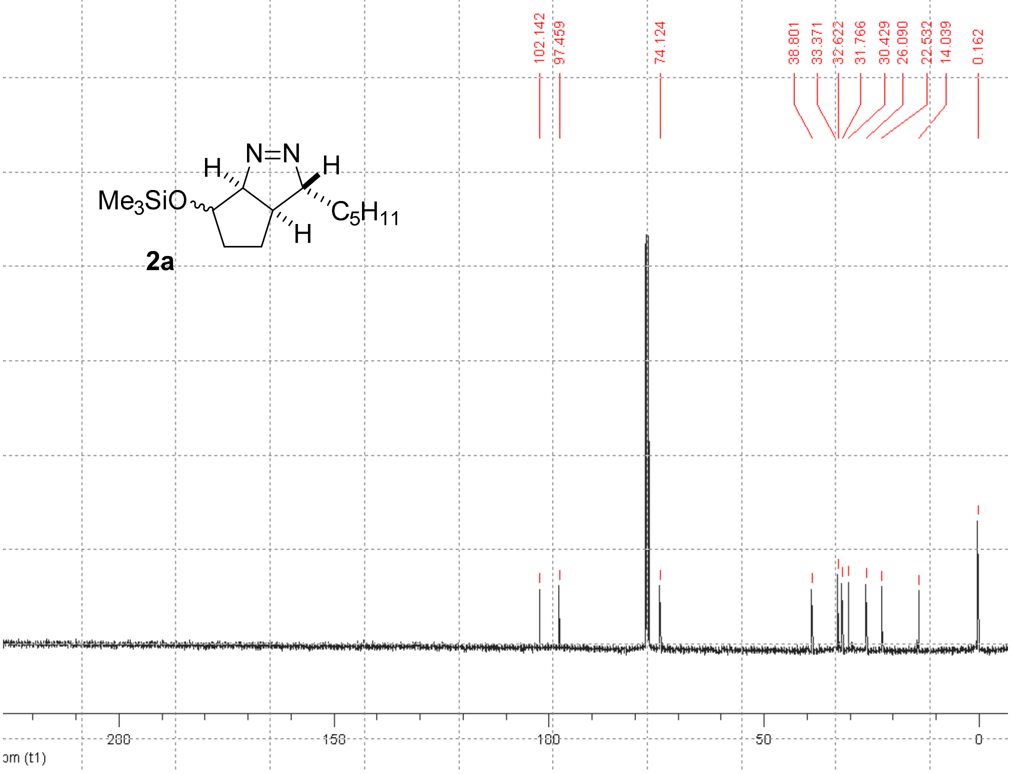

102.142
97.459
74.124
38.801
33.371
32.622
31.766
30.429
26.090
22.532
14.039
0.162
N=N
H
H
Me3SiO
C5H11
H
2a
200
150
100
50
0
ppm (t1)

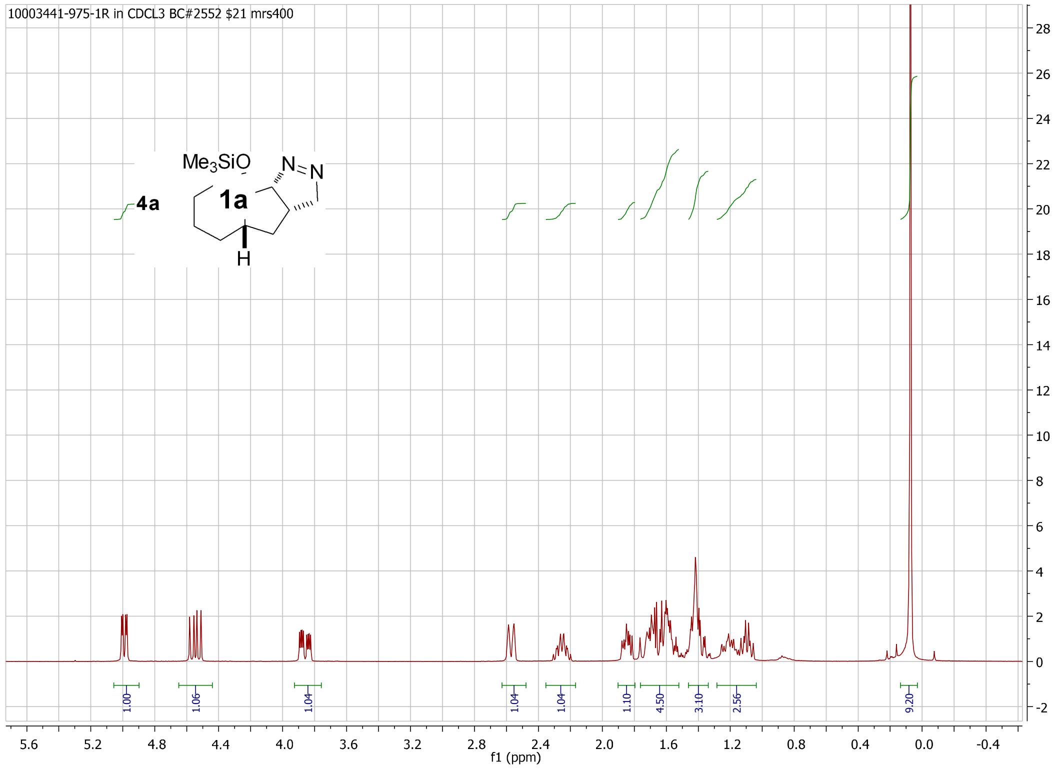
10003441-975-1R in CDCL3 BC#2552 $21 mrs400
Me3SiO
N=N
4a
1a
H
1.00
1.06
1.04
1.04
1.04
1.10
4.50
3.10
2.56
9.20
f1 (ppm)

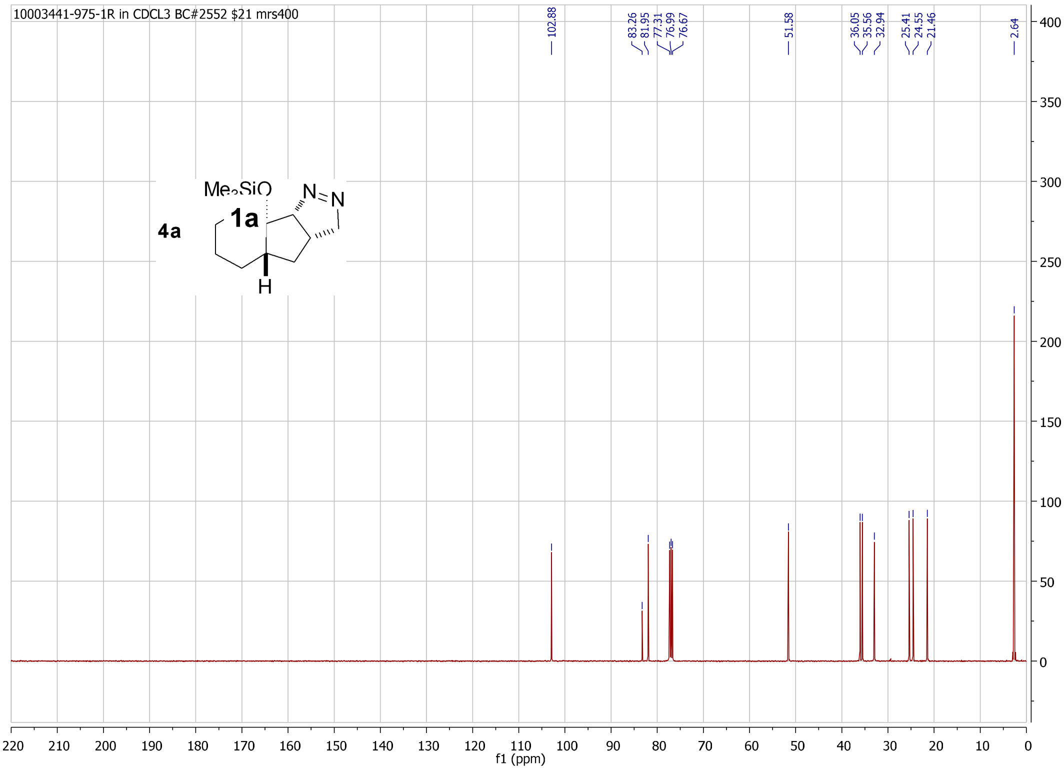
10003441-975-1R in CDCL3 BC#2552 $21 mrs400
102.88
83.26
81.95
77.31
76.99
76.67
51.58
36.05
35.56
32.94
25.41
24.55
21.46
2.64
Me3SiO
N=N
4a
1a
H
f1 (ppm)

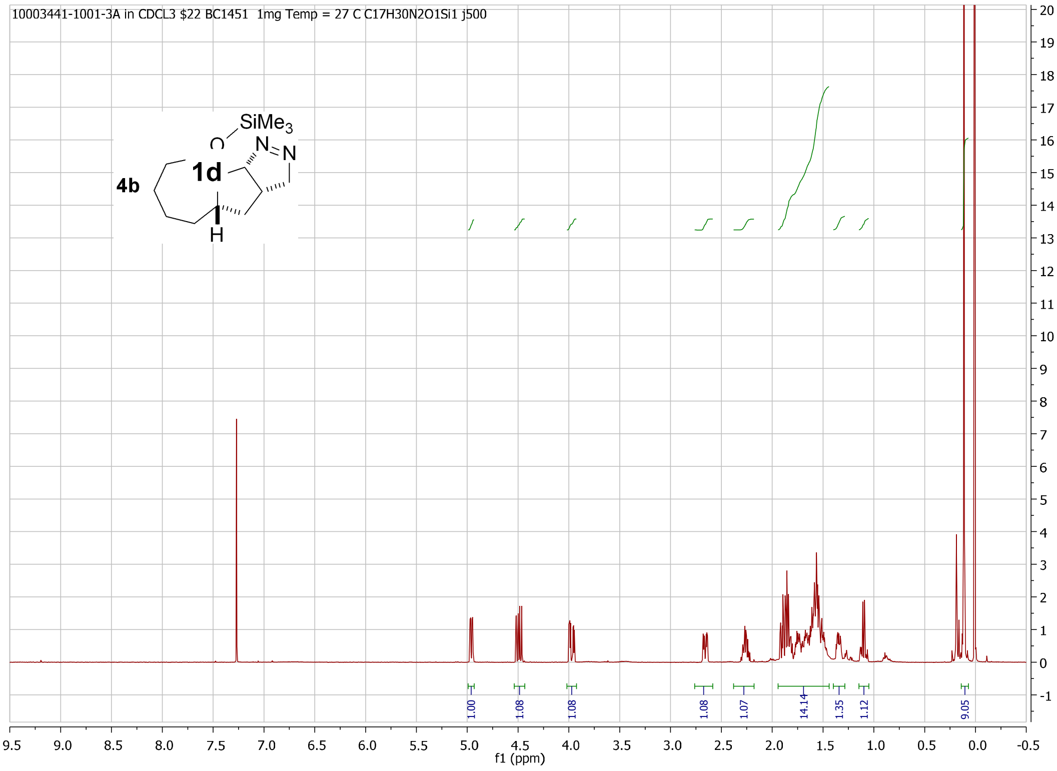
10003441-1001-3A in CDCL3 $22 BC1451 1mg Temp = 27 C C17H30N2O1Si1 j500
SiMe3
O
N
N
1d
4b
H
1.00
1.08
1.08
1.08
1.07
14.14
1.35
1.12
9.05
f1 (ppm)

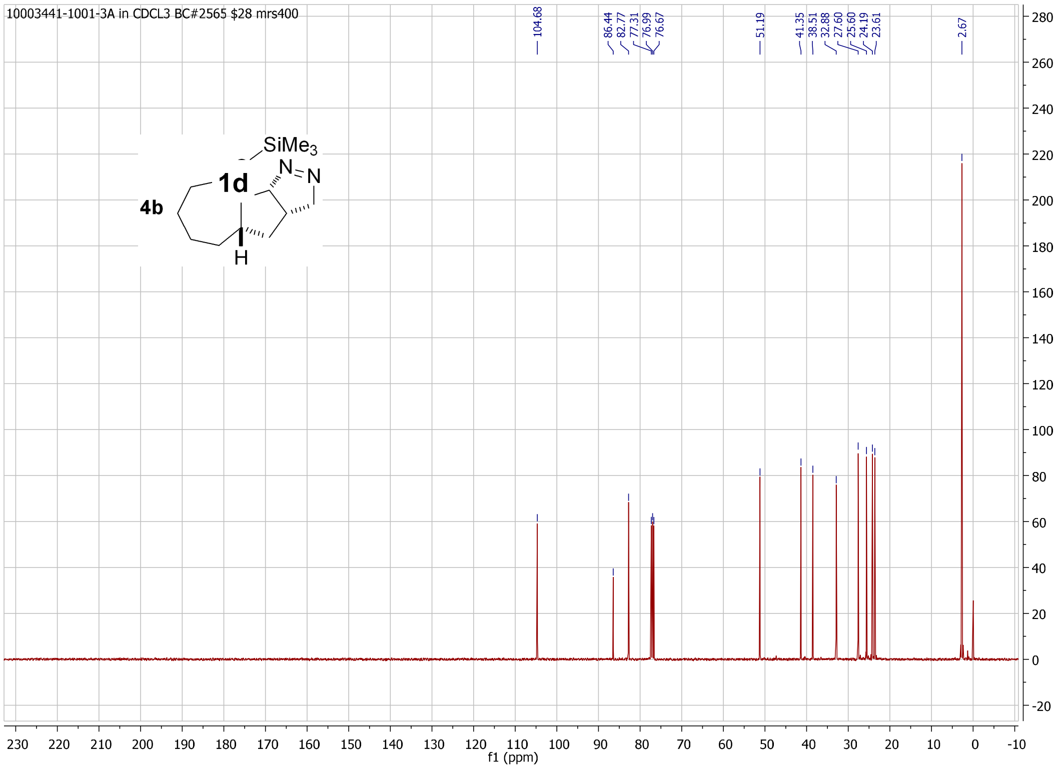
10003441-1001-3A in CDCL3 BC#2565 $28 mrs400
104.68
86.44
82.77
77.31
76.99
76.67
51.19
41.35
38.51
32.88
27.60
25.60
24.19
23.61
2.67
SiMe3
N
N
1d
4b
H
f1 (ppm)

6. ORTEP Diagrams

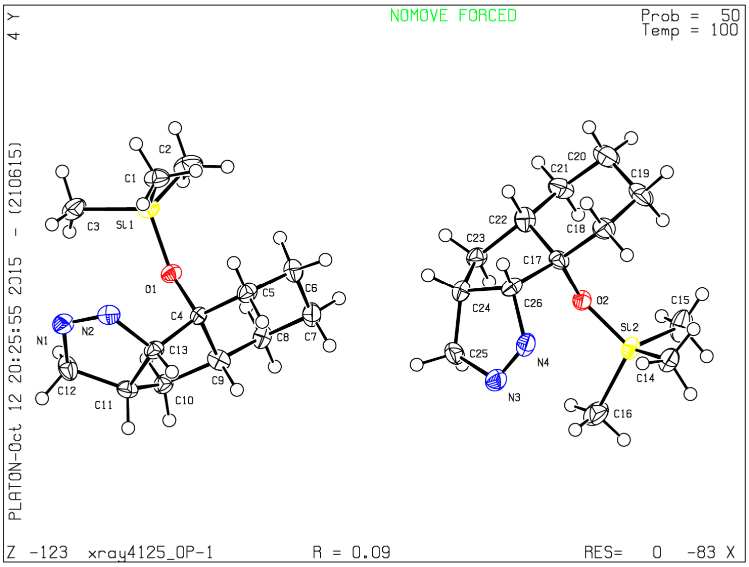

1a

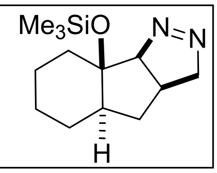

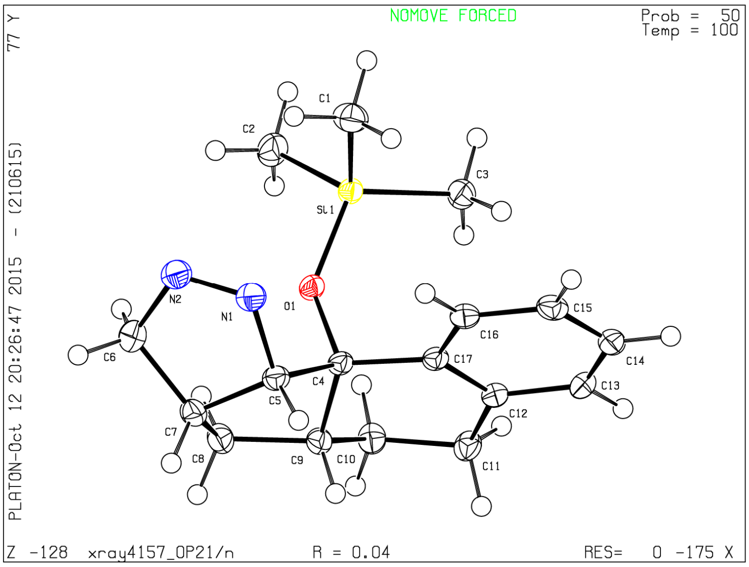

1b

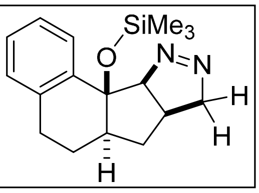

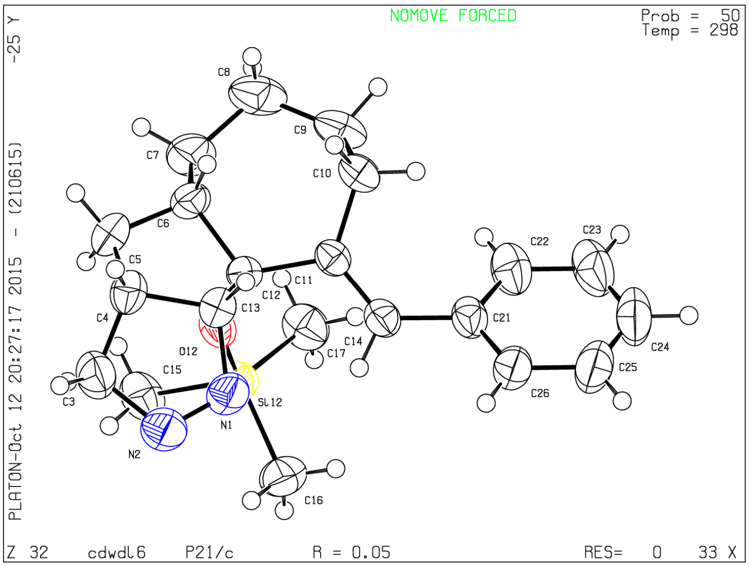

1e

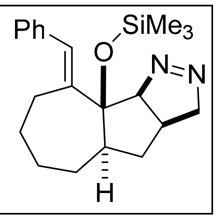

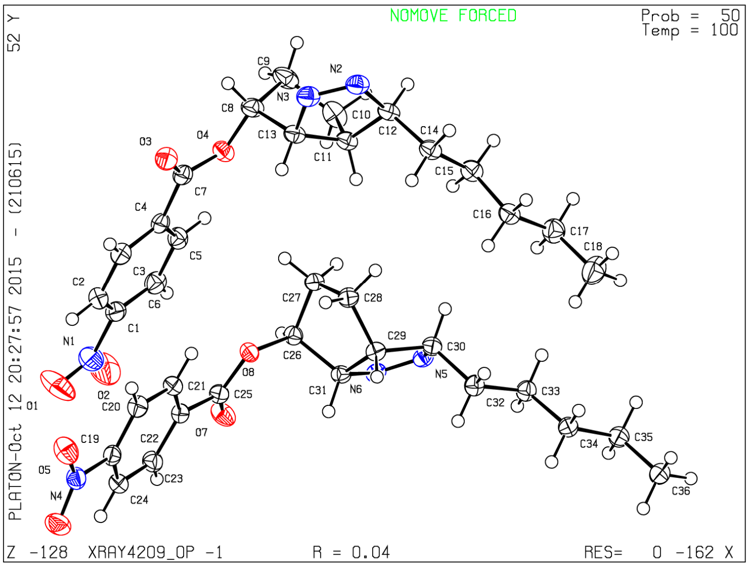

PNB-2a

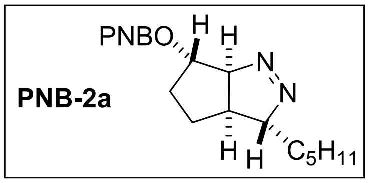

7. Geometric TSs with Bond Lengths

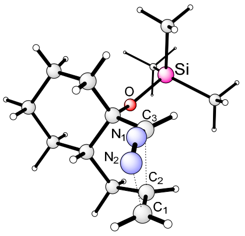

anti-TS-1b C_1-N_1 = 2.332 C_2-C_3 = 2.227

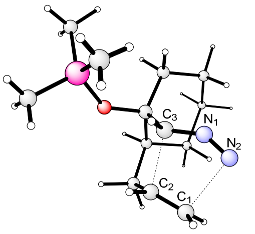

anti-TS-1b' C_1-N_1 = 2.391 C_2-C_3 = 2.198

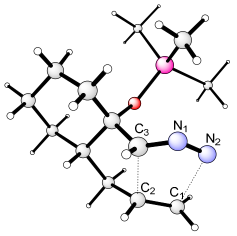

syn-TS-1b C_1-N_1 = 2.353 C_2-C_3 = 2.196

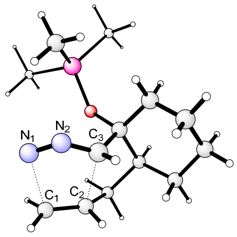

syn-TS-1b' C_1-N_1 = 2.367 C_2-C_3 = 2.188

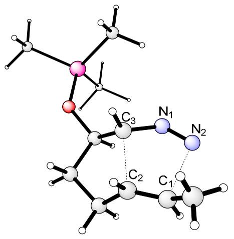

anti-TS-2a C_1-N_1 = 2.359 C_2-C_3 = 2.186

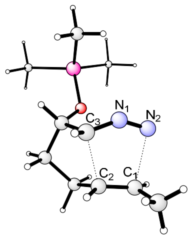

syn-TS-2a C_1-N_1 = 2.359 C_2-C_3 = 2.212

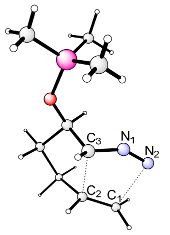

anti-TS-2b C_1-N_1 = 2.357 C_2-C_3 = 2.189

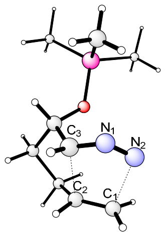

syn-TS-2b C_1-N_1 = 2.364 C_2-C_3 = 2.191

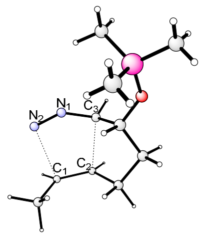

anti-TS-2c C_1-N_1 = 2.364 C_2-C_3 = 2.201

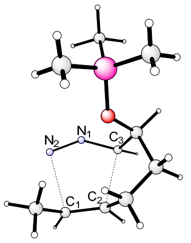

syn-TS-2c C_1-N_1 = 2.379 C_2-C_3 = 2.205

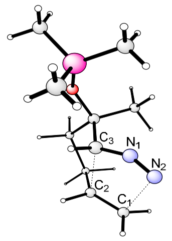

anti-TS-3a C_1-N_1 = 2.371 C_2-C_3 = 2.175

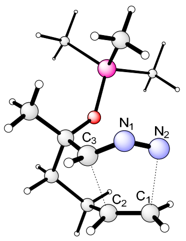

syn-TS-3a C_1-N_1 = 2.368 C_2-C_3 = 2.186
